# The availability and affordability of orphan drugs for rare diseases in China

**DOI:** 10.1186/s13023-016-0392-4

**Published:** 2016-02-27

**Authors:** Shiwei Gong, Yingxiao Wang, Xiaoyun Pan, Liang Zhang, Rui Huang, Xin Chen, Juanjuan Hu, Yi Xu, Si Jin

**Affiliations:** Department of Pharmacy Business and Administration, School of Pharmacy, Tongji Medical College of Huazhong University of Science and Technology, Wuhan, 430030 Hubei China; Department of Pharmaceutical Systems and Policy, School of Pharmacy, West Virginia University, Morgantown, WV USA; School of Health and Medicine Management, Tongji Medical College of Huazhong University of Science and Technology, Wuhan, Hubei China; Department of Endocrinology, Institute of Geriatric medicine, Liyuan Hospital, Tongji Medical College of Huazhong University of Science and Technology, Wuhan, Hubei China

**Keywords:** Rare disease, Orphan drug, Availability, Price, Affordability, China

## Abstract

**Background:**

Orphan drugs are intended to treat, prevent or diagnose rare diseases. In recent years, China healthcare policy makers and patients have become increasingly concerned about orphan drug issues. However, very few studies have assessed the availability and affordability of orphan drugs for rare diseases in China. The aim of this study was to provide an overview of the availability and affordability of orphan drugs in China and to make suggestions to improve patient access to orphan drugs.

**Methods:**

Two components of the availability of orphan drugs were examined. Market availability was assessed by the extent to which orphan drugs were marketed in China with a comparison to orphan drugs in international markets, such as the U.S., EU and Japan. We conducted surveys and collected data from 24 tertiary public hospitals in China to measure hospital-level availability of orphan drugs. The affordability of orphan drugs was calculated using hospital dispensary prices and was expressed as days of average daily income required for the cost of a course of treatment. Affordability was also analyzed under the Chinese basic medical insurance system.

**Results:**

Orphan drugs approved in the U.S., EU and Japan had 37.8 %, 24.6 % and 52.4 % market availability in China, respectively. Median availability of 31 orphan drugs surveyed at the 24 tertiary public hospitals was 20.8 % (very low). Within a periodic treatment course, the average treatment cost of 23 orphan drugs is approximately 4, 843. 5 USD, which equates to 505.6 days of per capita net income for an urban resident with a middle income (187.4 days for a high-income urban resident) or 1,582.8 days’s income for a rural resident with a middle income (657.2 days for a high-income rural resident). Except for homoharringtonine, 22 orphan drugs for 14 rare diseases were unaffordable for the most of residents in China. With 5 % out-of-pocket expenses, only three generics could be afforded by middle-income residents, whereas seven drugs for high-income urban residents.

**Conclusions:**

The Chinese government can take more responsibility for improving the availability and affordability of orphan drugs through setting up incentive policies and public platforms for sharing of orphan drug information. Control of the high price of orphan drugs, combined with a joint funding model from both government and private enterprise can efficiently reduce the economic burden of affected patients in China.

**Electronic supplementary material:**

The online version of this article (doi:10.1186/s13023-016-0392-4) contains supplementary material, which is available to authorized users.

## Background

Rare diseases are diseases with low prevalence that affect a very small proportion of the population [1]. These diseases are often serious, chronically debilitating, life-threatening or life-limiting [2] . Currently, there is no universally accepted definition of a rare disease. In the United States of America (U.S.), rare diseases are defined as those diseases that affect less than 200,000 persons (prevalence of < 0.64 ‰), whereas the definition shifts to an affected population of less than 50,000 in Japan (prevalence of < 0.39 ‰) and less than 2,000 in Australia (prevalence of < 0.1 ‰). In the European Union (EU), rare diseases are defined as life-threatening or chronic-debilitating conditions that affect less than 5 in 10,000 persons. Generally, the prevalence rate of rare diseases varies from about 1 to 6.4 in 10,000. Compared to the U.S., Japan, EU and Australia, the World Health Organization (WHO) defines a rare disease based on a higher prevalence: less than 6.5–10 in 10,000 [1]. Although any given rare disease affects a small percentage of the population, cumulatively, these diseases have a large impact as greater than 7, 000 different rare diseases have been reported [2]. The total number of patients with rare diseases is estimated to be approximately 30 million in Europe and 25 million in North America [3]. According to the definition of rare diseases recommended by WHO, over 10 million patients have rare diseases in China, estimated from a total population of at least 1.3 billion [4].

A drug that has been developed with the intent to diagnose, prevent or treat a rare disease may receive orphan drug status in the U.S., Japan, EU and Australia [5]. It is often very difficult for drug companies to quickly recover the research and development costs of orphan drugs. In 1983, the first incentive legislation, Orphan Drug Act (ODA) was passed in the U.S.. Currently, the ODA has been created not only to offer hope for patients with rare diseases, but also to foster a new mechanism of personalized drug and biotechnology development [6]. However, orphan drugs are characterized by both low market share and high price due to a low cost sharing population, which usually leads to limited access to affected patients. For example, the U.S. pharmacy retail prices of Novoseven (5 mg/1 kit), Gleevec (400 mg/30 tablets) were over $9,200 and $7,000 in 2013, respectively [7]. Therefore, it is very challenging to improve the affordability of these drugs for patients. Due to the large number of rare disease-affected patients in China, the shortage of orphan drugs for these patients has become a major health problem. Patients with rare diseases in China face a double dilemma [8].

Availability and affordability are the two main criteria to assess whether patients can receive timely, adequate and efficient treatment. Some studies have examined the availability and affordability of essential medicines for common diseases using the standardized methodology developed by the WHO and Health Action International (HAI) in 2008 [9–12]. Compared to orphan drugs, essential medicines are “those that satisfy the priority health care needs of the population” [13]. In 1977, the first “WHO Model List of Essential Medicine” identified 208 individual drugs which could provide safe, cost-effective, affordable and time-efficient treatments for common diseases. So far, the 19th WHO Model List released 409 essential drugs including innovative treatments for hepatitis C, breast cancer and leukemia, as well as multi-drug resistant tuberculosis, *etc*. [14]. However, from the view of disease prevalence, everyone has the potential to suffer from a rare disease. Effective and safe orphan drugs are of the utmost importance, and are indispensable for patients with rare diseases. Most recent studies have focused on international comparisons of orphan drug policies and regulations [15]. There has been a paucity of literature on the availability and affordability of orphan drugs for rare diseases in China. The objective of this study is to assess the availability and affordability of orphan drugs in China.

## Methods

### Data sources

We conducted two types of data collection. Firstly, we obtained three databases of orphan drug designations and approvals from the U.S. Food and Drug Administration (FDA) [16], European Medicines Agency (EMA) [17] and Japan Ministry of Health, Labour and Welfare (MHLW) [18]. According to these lists of orphan drugs, we confirmed their marketing authorization in China based on the drug approval database from the China Food and Drug Administration (CFDA) [19]. Using the Anatomical Therapeutic Chemical (ATC) classification system of the WHO to identify the ATC code for each orphan drug [20], we confirmed the ATC codes and calculated the category number of all orphan drugs marketed in U.S., EU, Japan and China. If the WHO had not defined the ATC code for an orphan drug, we confirmed the ATC type based on the type of disease treated by the orphan drug. We then compared the status of orphan drugs for 14 rare diseases in the U.S., EU, Japan and China.

We also developed a survey questionnaire to collect the price and availability information of each orphan drug ( both “brand name” and generic drugs) in each medical facility (such as tertiary public hospitals) based on the methodology of the WHO/HAI manual (2008). Data were collected by trained graduate students. These students were supervised by survey managers and the principal investigator. Responses on the survey forms were recorded carefully on the day of data collection.

### Selection of diseases and medications

Based on the rare diseases with the main orphan drug approvals in U.S., Japan and EU countries, we selected 14 life-threatening and serious diseases that could be considered as rare diseases in China according to WHO definition [1, 5, 21]. According to the annual new drug review reports of FDA, EMA, 14 rare diseases have orphan drugs with substantial therapeutic value. Twenty-four orphan drugs can be used to treat these 14 rare diseases, which cover cancers, blood diseases, cardiovascular diseases, metabolic diseases, endocrine diseases, neurologic diseases and respiratory diseases. These orphan drugs are imiglucerase, sapropterin dihydrochloride, recombinant coagulation factor VIII, coagulation factor VIIa, human coagulation factor VIII, human prothrombin complex concentrate, bosentan, Iloprost, ambrisentan, recombinant human growth hormone, busulfan, teniposide, mitoxantrone, imatinib, dasatinib, nilotinib, meisoindigo, arsenious acid, homoharringtonine, rituximab, sorafenib, danazol, riluzole and poractant alfa. The 24 types of orphan drugs included 22 brand name drugs and 9 generic drugs. (Additional file 1: Table S1 shows the names of drugs surveyed and their indications.)

According to the definition of WHO/HAI (2008), a brand name drug is a patented, pharmaceutical product that has been authorized for marketing worldwide. A generic drug is a pharmaceutical product that is “identical--or bioequivalent--to a brand name drug in dosage form, safety, strength, route of administration, quality, performance characteristics and intended use*”* [9, 22].

### Survey and selection of medical facilities

We conducted a pre-survey of 14 public hospital pharmacies and 28 private pharmacies in Wuhan, Hubei Province (per capita GDP in Wuhan is close to the median per capita GDP in China). We surveyed both hospital dispensary prices and pharmacy retail prices in 14 public hospitals and 28 private pharmacies in Wuhan. Our pre-survey results showed that most of the orphan drugs were only available in tertiary public hospitals, and were rarely found in secondary and primary hospitals. Only one orphan drug (Busulfan) was found in 3 out of 28 private pharmacies. Due to the unavailability of most orphan drugs, secondary or primary hospitals and private pharmacies were excluded from our final survey. Therefore, we would expect to find orphan drugs at large and well-known hospitals, such as public tertiary hospitals.

We surveyed the availability and prices of 24 orphan drugs in 24 tertiary public hospitals in China. These orphan drugs covered 135 drug package sizes during our survey time period (March 2012 to August 2012) according to the WHO/HAI methodology (2008). (Additional file 2: Table S2 shows the questionnaire on the availability and prices of orphan drugs for several serious rare diseases in the surveyed hospitals). The 24 hospitals were selected from 12 provinces and cities in the eastern, central and western regions of China, including Qinghai, Xinjiang, Sichuan, Yunnan (western region), Hubei, Anhui, Hunan (central region), Fujian, Guangxi, Guangdong, Zhejiang, and Beijing (eastern region). As public hospitals in China have adopted a centralized pharmaceutical bidding and distribution system through local governments to purchase drugs, similar types of drugs were generally available at the same level of hospital in each province. Hence, we believe the 24 tertiary public hospitals are representative of all tertiary public hospitals nationwide.

### Ethics

The study was approved by the Ethics Committee of Tongji Medical College, Huazhong University of Science and Technology (No: IORG0003571). We informed all participants of the aims and plans of our study before the survey. All participants provided their written informed consent to participate in this study.

### Measures and analysis

#### Availability

We measured orphan drug availability at both the national and hospital level according to the WHO definition and determinants of drug availability [23]. The market availability of an orphan drug at the national level was defined as (i) numbers and types of approved orphan drugs in China, the U.S., EU and Japan; and (ii) the marketing situation of orphan drugs for 14 rare diseases to be authorized in China, the U.S.**,** EU and Japan**.**

The hospital-level availability of orphan drugs was measured as the percentage of all surveyed public hospitals that could supply a particular orphan drug on the day of data collection, based on WHO/HAI methodology (2008).

According to the literature [11, 23], the following criteria were used to describe the availability of orphan drugs:

- Absent, 0 % of facilities: these orphan drugs were not found in any facilities surveyed;
- Very low, < 30 % of facilities: these orphan drugs were very difficult to find;
- Low, 30–49 % of facilities: these orphan drugs were somewhat difficult to find;
- Fairly high, 50–80 % of facilities: these orphan drugs were available in many facilities;
- High, >80 % of facilities: these orphan drugs had good availability.

High availability of orphan drugs at the hospital level will help patients with rare diseases get timely and efficient treatment.

### Affordability

According to WHO/HAI standard [9] , affordability can be measured as the ability of residents to afford a treatment course based on daily wages of the lowest-paid unskilled government worker. Due to the lack of official data on daily wages of the lowest-paid workers, we used disposable income per capita of urban residents and net income per capita of rural residents as proxy values [11]. Considering the high price of some orphan drugs, we selected two different income levels to reflect treatment affordability for urban and rural residents. The two income levels represent the average income of all residents and the average income of the highest income residents, which represent the middle-income and the high-income populations, respectively. In 2011, disposable daily income per capita of all urban residents was 59.75 RMB (9.58 USD/day) versus 161.21 RMB (25.84 USD / day) for high income urban residents; daily net income per capita of all rural residents was 19.12 RMB per day (3.06 USD / day) versus 45.98 RMB per day (7.37 USD / day) for high income rural residents in China [24]. If the cost of a course of treatment of an orphan drug is one day’s income or less, it is considered affordable and vice versa. Cost for a treatment course is denoted in Eq. (1).

1 $$ \mathrm{The}\ \mathrm{cost}\ \mathrm{of}\ \mathrm{a}\mathrm{n}\ \mathrm{orphan}\ \mathrm{drug}\ \mathrm{treatment}\ \mathrm{course} = \mathrm{Median}\ \mathrm{price}\ \mathrm{of}\ \mathrm{unit}\ \mathrm{dose}\ \mathrm{of}\ \mathrm{drug}\ \mathrm{reported}\ \mathrm{b}\mathrm{y}\ \mathrm{facilities} \times \mathrm{Drug}\ \mathrm{daily}\ \mathrm{dose} \times \mathrm{Days}\ \mathrm{of}\ \mathrm{a}\ \mathrm{treatment}\ \mathrm{course} $$

The median price of unit dose is the median drug dispensary price per unit dose. The median drug dispensary price was not calculated until data had been collected from at least three dispensaries among the 24 public tertiary hospitals surveyed (Additional file 3: Table S3 shows the median price of each orphan drug from the surveyed results of 24 public hospitals). We used the clinical periodic treatment course of every orphan drug as the base-case analysis (Additional file 4: Table S4 shows how the drug daily dose and treatment course was calculated). We estimated the duration of a periodic treatment course for each orphan drug based on the package insert information, literature review and expert medical opinions. We defined four weeks as a course for chronic and rare conditions, such as PAH, GHD, HAE, CML, ALL, AML, APL, NHL, RCC, ALS, *etc*. The WHO defines the duration of a treatment course as 7 days for an acute indication and 30 days for a chronic indication. We used the WHO’s definition as the sensitivity analysis. WHO also defines the Defined Daily Dose (DDD) for some orphan drugs, which refers to the average daily dose of a drug prescribed for its major indication in adults. There are five orphan drugs defined as DDD by WHO, including imiglucerase 300U, bosentan 250 mg, iloprost 50 ug, danazol 600 mg, riluzole 100 mg [20]. However, for most orphan drugs surveyed, WHO does not provide information on the DDD. We obtained the daily dose information from the packet insert, medical literature and expert clinical opinions. DDD varies by the patients’ exact indication, body weight and stage of disease. A maintenance dosage regimen recommended in the manufacturer’s summary of product characteristics (SPC) was used. If a disease has different severity levels, we chose the dose for moderate severity.

## Results

### Market availability of orphan drugs in China

Table 1 compares the number of orphan drug designations and approvals in U.S., EU, Japan and China. For the 315 unique orphan drugs approved in the U.S. from 1983 to 2012, 119 received marketing approval in China. The market availability rate in China was, therefore, 37.8 %. Among 65 unique orphan drugs that were approved for marketing in the EU between 2000 and 2012, market availability in China was only 24.6 %. Among the 145 orphan drugs that were approved for marketing in Japan from 1993 to 2012, market availability in China was 52.4 %. In total, of the 408 orphan drugs approved worldwide, 165 drugs were approved for marketing in China (40.4 %). Of the 165 drugs approved in China, 114 (69. 1 %) were manufactured by the domestic pharmaceutical producers in China. (Additional file 5: Table S5 lists the situation of 165 unique orphan drugs marketed in China)

**Table 1:** The number of orphan drug designations and approvals in U.S., EU, Japan and China

Regions (Time range)	Number of orphan drug designations	Number of orphan drug approvals for marketing	Number of unique orphan drugs approved for marketing^a^ (A)	Number of unique orphan drugs marketed in China^a^ (B)	Market availability rate in China [(B)/(A) × 100 %]
U.S. (1983–2012.12.31)	2741	425^b^	315	119	37.8
EU (2000–2012.12.31)	1098	65	65	16	24.6
Japan (1993–2012.12.31)	300	185^c^	145	76	52.4
Total	4132	675	525	211	40.2
			408^a^	165^a^	40.4

^a^:A unique drug may have multiple approvals for different rare indications. ^b^Not excluding withdrawals of designations and approvals of four orphan drugs. ^c^Excluding revoked products

Table 2 compares the numbers of ATC therapeutic categories of orphan drug approvals in U.S., EU, Japan and China. As a result, the three most common types of orphan drugs marketed in four regions are antineoplastic and immunomodulating agents, anti-infectives and drugs acting on the digestive and metabolic systems. Rare cancer was the most common therapeutic area and 80 orphan drugs were used to treat rare cancers. Among the antineoplastic drugs, 56.3 % are used to treat hematopoietic cancers, and 51.1 % of antineoplastic drugs for hematopoietic cancers have been marketed in China. The top three groups of drugs with the highest market availability rate in China are drugs acting on the genito-urinary system and sex hormones, anti-infectives for systemic use and the musculoskeletal system. However, there was a high CV percent (>25 %) among the different types of orphan drugs marketed in China.

**Table 2:** ATC Categories of orphan drugs marketed in the U.S., EU, Japan and China

ATC Category	Orphan drugs of China/U.S.		Orphan drugs of China/EU		Orphan drugs of China/Japan		Orphan drugs of China/U.S. + EU + Japan^b^	
	N	%	N	%	N	%	N	%
A-Alimentary tract and metabolism	6/42	14.3	1/12	8.3	6/19	31.6	10/43	23.3
B-Blood and blood forming organs	7/40	17.5	1/3	33.3	4/11	36.4	9/31	29.0
C-Cardiovascular system	6/9	66.7	1/5	20.0	4/7	57.1	6/13	46.2
D-Dermatologicals	1/4	25.0	0/0	-	0/0	-	1/4	25
G-Genito-urinary system and sex hormones	8/10	80.0	0/1	0	1/2	50	7/10	70
H- Systemic hormonal preparations, excluding sex hormones and insulins	15/36	41.6	1/3	33.3	3/7	42.9	9/19	47.3
J- Anti-infectives for systemic use	12/32	37.5	0/2	0	28/43	65.1	32/58	55.2
L^a^- Antineoplastic and immunomodulating agents	53/136	39.0	10/28	35.7	40/66	60.6	55/124	44.4
L1 for blood cancers	20/45	44.4	6/13	46.2	12/18	66.7	23/45	51.1
L2 for other cancers	23/43	53.5	4/9	44.4	6/12	50.0	16/35	45.7
M- Musculoskeletal system	5/12	41.7	0/0	-	2/7	28.6	6/12	50.0
N- Nervous system	9/31	29.0	1/6	16.7	3/11	27.3	10/33	30.3
P- Antiparasitic products, insecticides and repellents	4/17	23.5	0/0	-	2/3	66.7	5/12	41.7
R- Respiratory system	2/10	20	0/2	0	1/3	33.3	2/9	22.2
S- Sensory organs	2/8	25	0/0	-	1/4	25	5/12	41.7
V- Various	9/34	26.5	1/3	66.7	1/2	50	8/28	28.6
TOTAL	139/421^c^	33.0	16/65	24.6	96/185	52.4	165/408	40.4
Coefficient of Variation(C.V.)	-	53.7	-	84.5	-	33.8	-	35.1

N: Number. L^a^: includes L1 and L2 drugs. ^b^: Number of unique orphan drugs approved for marketing in the U.S., EU and Japan. ^c^: Excluding withdrawals of designations and approvals of four orphan drugs

To determine the availability of orphan drugs in China, we further compared the year of launch of each drug with other countries. Among 165 orphan drugs approved in China, 142 drugs (86.1 %) were approved from 1986 to 2008 in the U.S., EU or Japan. The number of targeted orphan drugs for 14 rare diseases surveyed in the U.S., EU and Japan were 64, 17 and 29 respectively. In total, there were 71 orphan drugs which can be used to treat these 14 rare diseases. Out of 71 orphan drugs for 14 rare diseases, 35(49. 3 %) were not available in China at the time of the survey, for example, miglustat, icatibant, ecallantide. (Additional file 6: Table S6 shows the 35 unauthorized orphan drugs for 14 rare diseases in the China.)

As shown in Table 3, 36 out of 71 orphan drugs were available in China (marketing availability rate 50.7 %). However, 31 out of 36 orphan drugs (86.1 %) had a delayed market launch in China. The average time of delay to market authorization was 7.7 years. The shortest delay to market authorization was 1 year (Poractant alfa) and the longest delay was 19 years (Pegaspargase). Two orphan drugs were released in the same year in China, which is also the first country they were launched.

**Table 3:** Market availability status of orphan drugs for 14 rare diseases in the U.S., EU, Japan and China

ATC Code	Generic name (Brand name)	Indication	Year marketing authorization received				Delay of release in China (in years) Compared to the earliest time
			China	U.S.^a^	EU^a^	Japan^a^	
A16AB02	Imiglucerase (Cerezyme)^b^ P	GD	2008	1994	^d^	1998	14
A16AX07	Sapropterin Dihydrochloride (Kuvan)^b^ P	PKU	2010	2007	2008	--	3
B02BD02	Human coagulation factor VIII^e^	HEM	2002	-- D	--	--	--
B02BD02	Recombinant coagulation factor VIII (Kogenate FS)		2010	-- D	-- D	--	--
--	Human prothrombin complex concentrate (^e^, Kcentra)		2002	2013	--	--	−11
B02BD04	Coagulation factor IX ( Benefix)^c^		2012	1997	D, ^d^	2009	15
B02BD05	Coagulation factor VIIa ( Novoseven)		2010	1999	D, ^d^	2000	11
H01BA02	Desmopressin acetate spray (^e^, Stimste)		2001	1994	--	--	7
C02KX01	Bosentan (Tracleer)^b^	PAH	2011	2001	2002	2005	10
B01AC19	Beraprost sodium (Dorner)		2008	-- D	-- D	1999	9
B01AC11	Iloprost inhalational solution (Ventavis)^b^		2007	2004	2003	--	5
C02KX02	Ambrisentan (Volibris/Letairis)^b^		2010	2007	2008	2010	3
B01AC21	Treprostinil (Remodulin)^b^ P		2014	2002	--	--	12
G04BE08	Tadalafil (Cialis® / Adcirca®)		2009	2009	^d^	--	0
G03XA01	Danazol ^e^	HAE	2002	--	--	--	--
H01AC01	RDNA origin somatropin (^e^,Norditropin SimpleXx/Norditropin)	GHD	1999	2000	--	1997	2
H01AC01	Somatropin (Saizen)		2000	1996	--	--	4
H01AC01	Somatropin (Genotropin)		2000	1997	--	1997	3
H01AC01	Somatropin (Humatrope) P		2002	1987	--	--	15
--	Recombinant human growth hormone^e^		2006	--	^d^	--	--
L01AB01	Busulfan (Busulfex/Busilvex ) P	CML	2002	1999	2003	2006	3
L01XE01	Imatinib (^e^, Glivec/Gleevec)^b^ P		2010	2001	^d^	2001	9
L01XE06	Dasatinib (^e^, Sprycel)^b^ P		2011	2006	2006	2009	5
L01XE08	Nilotinib (Tasigna)^b^		2009	2007	2007	2009	2
--	Meisoindigo		2010	--	--	--	--
L01BB02	Mercaptopurine (^e^, Xaluprine)	ALL	2002	2014	2012	--	- 10
L01AA01	Cyclophosphamide (^e^, Endoxan)		2001	-- D	--	2003	- 2
L01CB02	Teniposide (^e^, Vumon)^b^ P		1999	1992	--	--	7
L01XX24	Pegaspargase (^e^, Oncaspar)^c^		2013	1994	--	--	19
L01XE01	Imatinib (^e^, Gleevec / Glivec)		2010	2006	^d^	2001	9
L01XE06	Dasatinib (^e^, Sprycel)		2011	2006	2006	2009	5
L01DB07	Mitoxantrone HCl (^e^, Novantrone)^b^	AML	2001	1987	--	--	14
L01DB06	Idarubicin HCl (^e^, Zavedos/Idamycin)^b^ P		2002	1990	--	--	12
L01BC01	Cytarabine (^e^, Cylocide N)		2000	--	--	2000	0
--	Homoharringtonine		2002	-- D	-- D	--	--
--	Arsenious Acid (Yitaida)	APL	2010	--	--	--	--
L01XX27	Arsenic Trioxide (^e^,Trisenox)^b^ P		2008	2000	^d^	--	8
L01XX14	Tretinoin (^e^,Vesanoid) P		2002	1995	--	1995	7
--	Homoharringtonine		2002	-- D	-- D	--	--
L01XC02	Rituximab (MabThera/Rituxan)^c^	NHL	2000	1997	^d^	2001	3
L01XE05	Sorafenib (Nexavar)^b^ P	RCC	2011	2005	2006	--	6
L01XC07	Bevacizumab (Avastin)		2012	2009	^d^	--	3
N07XX02	Riluzole (^e^, Rilutek)^b^ P	ALS	2010	1995	^d^	1998	15
R07AA	Poractant alfa (Curosurf)^b^	PIRDS	2000	1999	--	--	1
--	Calf pulmonary surfactant (Kelisu)		2010	--	--	--	--

^a^:Drugs were approved as an orphan drug; ^b^:NME, new molecular entity as defined by the FDA; ^c^:NBE, new biologic entity as defined by the FDA; D: Orphan drug designation authorized; P: The drug received FDA priority drug review. ^d^:The drug was intended for rare diseases in Europe with European market authorization without priority designation of orphan drug in Europe; ^e^:The drug had a generic equivalent in China

Futhermore, eight drugs for six rare diseases were marketed only in China***.*** Four out of eight drugs were firstly approved in China, including arsenious acid, homoharringtonine, meisoindigo, calf-pulmonary surfactant for injection. Table 3 also shows that 19 ophan drugs which were approved as a new molecular entity (NME) or new biological entity (NBE) by the FDA receieved marketing approval in China.

### The availability of orphan drugs at a hospital level

In China, drug procurement in tertiary public hospitals depends on the centralized pharmaceutical bidding system of provincial governments. In 2011, 120 out of 165 orphan drugs were covered by at least one government’s centralized bidding system (Additional file 5: Table S5 shows the number of procurement provinces for 120 orphan drugs). To further investigate the actual availability of orphan drugs in 2012, we surveyed and summarized data from 24 public tertiary hospitals that are representative of the national situation and presented the results in Table 4. Overall, the median availability rate of all orphan drugs surveyed was 20.8 %, with 18.9 % for brand name drugs and 45.8 % for generics. Four approved drugs including ambrisentan, somatropin, dasatinib and meisoindigo were not available in the hospitals surveyed. Nineteen orphan drugs (15 brand names, 5 generics) were found to be available in less than 50 % of all hospitals surveyed. Seven orphan drugs (3 brand names, 4 generics) were available in > 50 % of the surveyed hospitals.

**Table 4:** Availability of brand name and generic orphan drugs surveyed in 24 public tertiary hospitals in China

Availability	Range	Originator Brands (Brand Name)	Generics
Absent	0	Ambrisentan (Volibris), Somatropin (Genotropin), Dasatinib (Sprycel), Meisoindigo	None
Very low	<30 %	Imiglucerase (Cerezyme), Sapropterin dihydrochloride (Kuvan), Coagulation factor VIIa (Novoseven), Bosentan (Tracleer), Iloprost (Ventavis), Somatropin (Saizen), Somatropin (Humatrope), Busulfan(Busulfex), Imatinib Mesylate Cap (Glivec), Nilotinib (Tasigna), Riluzole ( Rilutek)	Teniposide, Riluzole tab^a^, Riluzole cap^a^
Low	30–49 %	Recombinant coagulation factor VIII (Kogenate FS), Imatinib mesylate Tab (Glivec), Arsenious acid(Yitaida), Sorafenib (Nexavar)	Human coagulation factor VIII, Danazol
Fairly high	50–80 %	Teniposide (Vumon), Rituximab (MabThera), Poractant alfa (Curosurf)	Human prothrombin complex concentrate, Recombinant human growth hormone, Mitoxantrone, Homoharringtonine
High	80 %	None	None

^a^:Tab: Tablets, Cap: Capsules

Of the 31 orphan drugs surveyed, 23 had pricing information and were available in at least three hospitals. The unit prices of branded and generic orphan drugs surveyed can be found in Additional file 2: Table S2.

### The affordability of orphan drugs

In 2011, the average unit procurement price of the 120 orphan drugs was between $0.00003 and $881.3, compared to $0.002 and $881.3 for the orphan drugs surveyed (Additional file 5: Table S5 shows the procurement price of the 120 orphan drugs). The average unit procurement price of 21 orphan drugs was lower than $0.002. Table 5 presents the hospital dispensary prices, cost of treatment and affordability of orphan drugs surveyed in 2012. Among the 23 orphan drugs with pricing information from at least three hospitals we analyzed, homoharringtonine is the only drug that would be affordable to an urban resident, with the treatment cost being less than one day’s income. The other 22 orphan drugs were unaffordable to either urban or rural resident with a middle income in China. Among those drugs that are unaffordable, NovoSeven is the most unaffordable, followed by Rituximab, Sorafenib tosylate, Imatinib and Bosentan.

**Table 5:** Affordability of orphan drugs in China

Generic name (Brand name)	Median Unit Price^A^ (USD)	Daily Dose	Duration of Treatment Course (days)	Total Course Cost (USD)	Days of per capita net income						Coverage of NBMI (Y/N)	Affordability (if 5 % OOP)	
					Urban Resident		Affordability	Rural Resident		Affordability			
												Ave (Y/N)	Urban High (Y/N)
					Ave^3^	High^4^	Y/N	Ave	High	Y/N			
Recombinant human coagulation factor VII (Kogenate FS)	0.85/IU	1750 IU	2	2961.5	309.1	114.6	N	967.8	401.8	N	Y, Part B	N	N
Recombinant human coagulation factor VIIa (NovoSeven)^B^	869.02/mg	50.4 mg	1	43798.7	4571.9	1695.0	N	14313.3	5942.8	N	N	N	N
Bosentan (Tracleer)	0.75/mg	250 mg	28	5272.4	550.4	204.0	N	1723.0	715.4	N	N	N	N
Iloprost (Ventavis)	4.38/ug	50ug	28	6129.5	639.8	237.2	N	2003.1	831.7	N	N	N	N
Somatropin (Saizen)	10.89/IU	7 IU	28	2134.3	222.8	82.6	N	697.5	289.6	N	N	N	N
Busulfan (Busulfex)	4.66/mg	224 mg	4	4178.5	436.2	161.7	N	1365.5	567.0	N	Y, Part B	N	N
Imatinib (Glivec)	0.32/mg	600 mg	28	5384.6	562.1	208.4	N	1759.7	730.6	N	N	N	N
Imatinib (Glivec)	0.32/mg	600 mg	28	5438.5	567.7	210.5	N	1777.3	737.9	N	N	N	N
Nilotinib (Tasigna)	0.26/mg	800 mg	28	5779.5	603.3	223.7	N	1888.7	784.2	N	N	N	N
Teniposide (Vumon)	0.56/mg	102 mg	5	286.1	29.9	11.1	N	93.5	38.8	N	N	N	**Y**
Arsenious acid (Yitaida)	2.32/mg	10 mg	28	650.6	67.9	25.2	N	212.6	88.3	N	Y, Part B^#^	N	N
Rituximab (MabThera)	6.1/mg	91.1 mg	28	15558.2	1624.0	602.1	N	5084.4	2111.0	N	N	N	N
Sorafenib tosylate (Nexavar)	0.33/mg	800 mg	28	7502.6	783.2	290.3	N	2451.8	1018.0	N	N	N	N
Poractant alfa (Curosurf)^C^	4.8/mg	525 mg	1	2456.7	256.4	95.1	N	802.8	333.3	N	Y, Part B	N	N
Human coagulation factor VII	0.3/IU	1750 IU	2	1065.7	111.2	41.2	N	348.3	144.6	N	Y, Part A	N	N
Human prothrombin complex concentrate	0.15 IU	3150 IU	2	969.2	101.2	37.5	N	316.7	131.5	N	Y, Part B^#^	N	N
Danazol	0.004/mg	600 mg	28	61.9	6.5	2.4	N	20.2	8.4	N	Y, Part B	Y	**Y**
Recombinant human growth hormone	3.7/IU	7 IU	28	725.6	75.7	28.1	N	237.1	98.5	N	Y, Part B	N	N
Teniposide	0.44/ mg	102 mg	5	223.1	23.3	8.6	N	72.9	30.3	N	Y, Part B	N	**Y**
Mitoxantrone	1.24/mg	10.2 mg	4	50.6	5.3	2.0	N	16.5	6.9	N	Y, Part B	Y	**Y**
Riluzole	0.16/mg	100 mg	28	453.2	47.3	17.5	N	148.1	61.5	N	N	N	**Y**
Riluzole	0.11/mg	100 mg	28	309.6	32.3	12.0	N	101.1	42.0	N	N	N	**Y**
Homoharringtonine	0.75/mg	2.5 mg	5	9.4	0.98	0.4	Y	3.1	1.3	N	Y, Part A^#^	Y	**Y**
Average	--	--	17.2	4843.5	505.6	187.4	--	1582.8	657.2	--	--	--	**--**

1.C/T: capsules/tablets, Inj: injection, Inh: inhalants. 2. In the calculations, we used the following average values: adult weight at 70 kg, children 15 kg, baby 1.5 kg; the body surface area at 1.7 m^2^. ^A^: Use the minimum specifications as the standards about the investigated drugs. Translated the price and took the median values as analysis objects; ^B^: Recommended dose is 90ug/kg, 8times a day. ^C^: Birth weight used was 1.5 kg. The first dose is 150 mg/kg; repeat dose is 100 mg/kg, only one day usage. 3. “Ave”: a resident with per capita net income of average income households. 4. “High”: a resident with per capita net income of highest income households. Y=Yes N=No. “NBMI”: National Basic Medical Insurance. “Part A” or “Part B” means a drug is covered by the Part A or Part B drug list of National Basic Medical Insurance. “OOP”: out-of-pocket expenses. “#”: a drug is a national essential medicine of China

Within a periodic course of treatment, the average treatment expenditure for the 23 orphan drugs was 4,843.5 USD, which is the equivalent of 505.6 days of per capita net income for an urban resident with a middle income (187.4 days for a high-income urban resident) and 1,582.8 days’s income for a rural resident with a middle income (657.2 days for a high-income rural resident). We also tested the change in affordability by using the WHO’s definition of a drug treatment course - either 7-days or 30-days. The results are quite unaffordable when the cost for a 7-day or 30-day course was analyzed. All 23 orphan drugs had higher cost and were unaffordable to either urban or rural resident with a middle income.

We further analyzed the data by accounting for the healthcare insurance system in China. Taking the Urban Employees’ Basic Medical Insurance Scheme (UEBMIS) as an example, the coverage range and reimbursement rate of UEBMIS is higher than the other two Chinese medical insurance schemes, namely the Urban Residents’ Basic Medical Insurance Scheme (URBMIS), and the New Rural Cooperative Medical Insurance Scheme (NRCMIS). UEBMIS covers the Part A and Part B drugs listed by the National Basic Medical Insurance [25].

For example, human coagulation factor VIII and homoharringtonine are covered in the Part A list, which are free for beneficiaries covered by UEBMIS. There are 9 orphan drugs covered in the Part B list. Generally, the beneficiaries of UEBMIS need to pay for Part B drugs at a certain out-of-pocket rate, which often ranges from 5 to 20 % in different provinces depending on local regulations. For essential drugs, they are covered by the three insurance schemes with the lower out-of-pocket rate. Of 165 orphan drugs, 22 were national essential drugs of China. There were three essential drugs among 31 orphan drugs surveyed. However, at a 5 % out-of-pocket rate of drug cost, only three generic drugs are affordable to residents with middle incomes, whereas seven orphan drugs for high-income urban residents.

## Discussion

In the present study, we have performed an international comparative analysis of orphan drug availability in the U.S., EU, Japanese and Chinese markets. Using the WHO/HAI methodology, we determined the availability, price and affordability of orphan drugs from surveyed information in 24 tertiary public hospitals in China.

There are four main findings from our research. Firstly, the market availability of orphan drugs was relatively low in China when compared to other countries. Among the 408 unique orphan drugs approved in the world, only 40.4 % of these are available in China. The majority of the orphan drugs marketed in China are used to treat cancers. The drug with the highest market availability rate in China acts on the genito-urinary system. The availability of orphan drugs marketed in China differs greatly depending on the disease being treated. Secondly, most orphan drugs authorized in other countries within the past 5 years have not been launched in China yet. Compared to the earliest launch time across the world, the average delay in market authorization of 31 orphan drugs for 14 rare diseases in China was 7.7 years. Thirdly, the median availability rate of 24 orphan drugs surveyed for 14 rare diseases in 24 top hospitals in China was low (20.8 %). The hospital availability rates of generics were higher than for branded orphan drugs. Finally, it can clearly be seen that there is a substantial economic burden for patients with rare diseases in China. The average treatment expenditure for the 23 orphan drugs equates to 187.4 days of per capita net income($ 25.84/day) for a high-income urban resident. Compared to essential drugs, Jiang’s study found the average treatment cost of 13 original brands and 16 lowest-priced generics for 11 common diseases equated to 3.27 days’ wages and 1 day’s wages ($ 4.70) in the public hospitals in Shaanxi Province in 2012 [12]. Both urban and rural residents with middle incomes could not afford the cost of most orphan drugs surveyed, let alone those with lower incomes. With 5 % out-of-pocket expenses, only seven drugs could be afforded by high-income urban residents.

Therefore, the reasons for low market availability, low public hospital availability and low affordability of orphan drugs in China deserve further discussion. Lack of R&D and supply incentive policies for orphan drugs are possible reasons that lead to low market availability. Under a market-oriented economy, due to a small market share of orphan drugs, most pharmaceutical manufacturers are not willing to invest in R&D and the production of orphan drugs without an incentive policy. In addition, since most rare diseases are caused by DNA mutations or are recessive genetic diseases [26], their diagnosis, treatment and drug R&D often depend on more funding and more advanced scientific instrumentation, which means a greater risk of investment and manufacturing. Currently, there is no incentive policy for R&D, production or the importation of orphan drugs in China. Our findings reveal a positive association between the number of marketed orphan drugs and the number of incentive policies in the U.S., EU and Japan.

There are further obstacles that contribute to the low availability rate of orphan drugs at the hospital level in China. Firstly, there is a lack of knowledge on rare diseases and training for medical doctors, *e.g.* diagnostic methods and clinical guidelines. Rare diseases are often under-diagnosed or misdiagnosed, especially in Chinese rural areas with less well-qualified medical professionals [27]. Under the current three-tier structure system of hospitals, primary care hospitals are still not performing optimal gatekeeping and referral roles [28]. Patients with rare diseases are more likely to miss out on effective and timely treatment with orphan drugs in the higher-level hospitals. Secondly, there are no public national or provincial networks for rare diseases or orphan drugs for the sharing of useful information in regards to these diseases, *e.g.* treatment information or supply information. Thirdly, in China, there are no specific procurement policies available for orphan drugs, no specifications for orphan drug use and no fixed suppliers of orphan drugs, *e.g.* wholesalers and manufacturers, as well as no uniform price control mechanisms. Fourthly, in hospital pharmacies, specific management organization and measures for orphan drugs are not currently established. Lastly, due to the special market attributes of orphan drugs, *e.g.* low market volume, low profit, low turnover rate, high price and high transaction cost (high search cost), hospitals tend to have limited interest in purchasing orphan drugs [29].

The low affordability of orphan drugs may be closely associated with many factors, *e.g.,* high drug price, tendency to use brand name drugs at hospitals, lack of insurance coverage, low reimbursement rates, and low income level for Chinese residents. These specific reasons could also be used to explain the different distributions of rare diseases and the availability of orphan drugs in different regional hospitals.

Our research emphasizes the need for future policy efforts to improve orphan drug availability, insurance coverage and to decrease drug prices in China. Further research is needed to clearly define rare diseases and orphan drugs using genetic testing and essential diagnostics to achieve accurate diagnosis of rare diseases [14, 30], to formulate a list of available orphan drugs and to develop incentive policies for orphan drugs. Future studies may focus on the feasibility and applicability of incentive policies for orphan drugs in China, based on the experience obtained in the regulation of orphan drugs in the US, EU, Australia, Japan and Singapore. Incentive policies should be directed towards those orphan drugs with low market or hospital availability, try to improve the efficiency of production and supply of orphan drugs and disseminate more information about orphan drugs and rare diseases to patients and doctors. More specific incentive systems are also needed to establish targeted subsidies for the production and supply of orphan drugs, which may include filing and reporting systems for orphan drug companies. Furthermore, import tariff exemption systems for orphan drugs and free public platforms for sharing orphan drug information from R&D to drug use for all the stakeholders also require development.

Given the low affordability of orphan drugs in China, more research is required to formulate policies to control the price of orphan drugs, to increase insurance coverage for rare diseases and to increase social relief funding. Previous studies have suggested several ways to lower orphan drug prices and costs. Barak et al. suggested lower market costs and enhancement of the close relationship among patients, medical personnel, key opinion leaders and advocacy groups may improve market access for orphan drugs [31]. Michel et al. found a greater number of available alternatives and increased competition from manufacturers may also decrease the prices of orphan drugs [32].

European countries have developed methods to control the prices of orphan drugs. Based on the results of an availability survey for orphan drugs in the EU and recommendations on the EU Pharmaceutical Forum of 2008, Eurordis suggested that an expert working group should be established to assess the relative effectiveness of each orphan drug, according to the added therapeutic value and negotiate an EU ex-factory reference price with manufacturers [33, 34]. The French Economic Committee signed an agreement with the pharmaceutical industry to restrict the annual cost of orphan drugs to €50,000 per patient. In Spain, a maximum price at which hospitals can buy orphan drugs is maintained at the national level [32].

As for the evaluation of pricing and reimbursement for new orphan drugs, Hughes-Wilson et al. proposed a new assessment system based on several criteria that can be used to evaluate newly developed orphan drugs at the time of pricing or reimbursement. These criteria include disease rarity, severity, the availability of other alternatives, the effectiveness of the new treatment, the cost of research undertaken by the developer, as well as other factors, such as manufacturing complexity and follow-up measures required [35].

Orphan drugs are often cost-ineffective at the priced levels compared to common drugs [36]. Some studies have suggested that cost-effectiveness analyses used to assess the reimbursement of orphan drugs also need to take into account health equity, societal values, therapeutic necessity, and the use of hierarchical methods in allocation of health resources [31, 37].

Several cost risk-sharing programs for rare diseases have been launched in European countries and the U.S., including patient access schemes, ring-fenced budgets and patient assistance programs [31]. Novel patient assistance and foundation programs are supported by patient organizations and drug manufacturers, such as NORD’s Hodgkin’s Lymphoma Co-Payment Assistance Program, and the Celgene Patient Support Program (Revlimid), among others [38]. Since 1987, NORD has administered over 380 patient assistance programs and given out $56 million worth of drugs for free, as well as offering co-payment assistance [39].

At present, in China, eight assistance programs for donated drugs have been developed by the China Charity Federation and drug manufacturers, and includes drugs such as Glivec ®, Tasigna ®, Nexavar ®, Tracleer ®, Cerezyme ®, Exjade ®, Iressa ® and Tarceva ®. In 2012, the Chinese government launched a new insurance scheme for all residents that deals with serious illnesses. This pilot scheme benefits patients with 20 serious and life-threatening diseases including childhood leukemia, breast cancer, cervical cancer, esophageal cancer, colon and colorectal cancer, CML, and HEM, among others. In 2013, imatinib mesylate and dasatinib obtained marketing authorization as generic drugs from the CFDA, using a special review and approval procedure [40]. On February 22, 2013, the CFDA also released a new notice on establishing an accelerated approval process for new drugs and a priority review of generic drugs for rare diseases. As described above, the Chinese government is paying more attention to the issue of orphan drugs. Therefore, it is necessary that future policies can assure a negotiated price for orphan drug with the pharmaceutical companies, encourage marketing approvals of generics with lower prices and establish a joint funding model that includes government funding for specific medical insurance and social or private capital for patient assistance programs, so as to lower the drug price and enhance the affordability of orphan drugs.

### Limitations

The availability of orphan drugs was measured at specific health facilities on the day of data collection. This measure of availability may not accurately capture the availability of medicines in the hospitals. The hospitals surveyed may normally have a product in stock or have a purchase contract with a drug company. However, they may have run out of the drug on the day of the survey.

We measured affordability based on the average and high income level of residents, which may not reflect affordability for residents with lower incomes. Moreover, our measure of affordability does not take into account other diagnostic or treatment costs. The true cost of health care for patients with rare diseases may have been underestimated.

In this study, 31 orphan drugs were evaluated with $0.002 to $881.3 unit procurement price. However, some common and cheap drugs with indications for rare diseases that had a unit procurement price below $0.002, were not analyzed for their availability and affordability. Therefore, the whole availability and affordability of orphan drugs may be underestimated to some degree in China.

## Conclusions

The article reveals that both the availability and affordability of orphan drugs in China are low. However, a good medication should be both available and affordable. In the absence of either factor, the drug is of little use to patients. In this context, the Chinese government can take further responsibility for improving the availability and affordability of orphan drugs through setting up incentive policies and public platforms for the sharing of orphan drug information. Control of the price of orphan drugs and a joint funding model from both government and private enterprise can efficiently reduce the economic burden of affected patients in China.

## Availability of supporting data

The data set supporting the results of this article are included within the article and its additional files.

## Additional files

Additional file 1: Table S1. List of 31 orphan drugs surveyed in 24 public tertiary hospitals in China. (DOC 46 kb) Additional file 2: Table S2. The questionnaire on the availability and prices of orphan drugs for several serious rare diseases in hospitals. (DOC 248 kb) Additional file 3: Table S3. The unit prices of brand name and generic orphan drugs surveyed in 24 public tertiary hospitals in China. (DOC 53 kb) Additional file 4: Table S4. The daily dose and course of treatment for the surveyed orphan drugs. (DOC 52 kb) Additional file 5: Table S5. List and general situation of 165 unique orphan drugs marketed in China. (DOC 316 kb) Additional file 6: Table S6. Unauthorized list of 35 orphan drugs for 14 rare diseases in the China market. (DOC 59 kb)

## Abbreviations

ALL: Acute Lymphoblastic Leukemia
ALS: Amyotrophic Lateral Sclerosis
AML: Acute Myeloid or Myelogenous Leukemia
APL: Acute Promyelocytic Leukemia
CML: Chronic Myeloid or Myelogenous Leukemia
GD: Gaucher’s Disease
GHD: Growth Hormone Deficiency
HAE: Hereditary Angioedema
HEM: Hemophilia
NBE: New Biologic Entity
NHL: Non-Hodgkin’s Lymphoma
NME: New Molecular Entity
PAH: Pulmonary Arterial Hypertension
PIRDS: Respiratory Distress Syndrome in Premature Infants
PKU: Phenylketonuria
RCC: Renal Cell Carcinoma
