# Supplementary material for: The availability and affordability of orphan drugs for rare diseases in China
Source: Orphanet J Rare Dis. 2016 Feb 27;11:20. doi: 10.1186/s13023-016-0392-4 (PMC4769558; doi:10.1186/s13023-016-0392-4)
Supplement: Additional file 1: Table S1. — List of 31 orphan drugs surveyed in 24 public tertiary hospitals in China. (DOC 46 kb) [file 13023_2016_392_MOESM1_ESM.doc]

**Additional file 1:** Table S1 List of 31 orphan drugs surveyed in 24 public tertiary hospitals in China

| NO | Generic name | Brand Name | Main Indication |
| --- | --- | --- | --- |
| 1 | Imiglucerase | Cerezyme | Gaucher's Disease(GD) |
| 2 | Sapropterin dihydrochloride tablets | Kuvan | Phenylketonuria(PKU) |
| 3 | Human coagulation factor VIII | -- | Hemophilia(HEM) |
| 4 | Recombinant coagulation factor VIII | Kogenate FS |
| 5 | Prothrombin complex concentrate(Human) | -- |
| 6 | Coagulation factor VIIa | Novoseven |
| 7 | Bosentan tablets | Tracleer | Pulmonary Arterial Hypertension (PAH) |
| 8 | Iloprost inhalational solution | Ventavis |
| 9 | Ambrisentan tablets | Volibris |
| 10 | Somatropin | Saizen | Growth Hormone Deficiency(GHD) |
| 11 | Somatropin | Genotropin |
| 12 | Somatropin | Humatrope |
| 13 | Recombinant human growth hormone | -- |
| 14 | Busulfan | Busulfex | Chronic Myeloid or Myelogenous Leukemia(CML) |
| 15 | Imatinib capsules | Glivec |
| 16 | Imatinib tablets | Glivec |
| 17 | Dasatinib tablets | Sprycel |
| 18 | Nilotinib capsules | Tasigna |
| 19 | Meisoindigo tablets | -- |
| 20 | Teniposide | Vumon | Acute Lymphoblastic Leukemia(ALL) |
| 21 | Teniposide | -- |
| 22 | Mitoxantrone hydrochlorid | -- | Acute Myeloid or Myelogenous Leukemia(AML) |
| 23 | Homoharringtonine | -- |
| 24 | Arsenious Acid | Yitaida | Acute Promyelocytic Leukemia(APL) |
| 25 | Rituximab | MabThera | Non-Hodgkin's Lymphoma(NHL) |
| 26 | Sorafenib tablets | Nexavar | Renal Cell Carcinoma(RCC) |
| 27 | Danazol capsules | -- | Hereditary Angioedema(HAE) |
| 28 | Riluzole tablets | Rilutek | Amyotrophic Lateral Sclerosis (ALS) |
| 29 | Riluzole capsules | -- |
| 30 | Riluzole tablets | -- |
| 31 | Poractant Alfa | Curosurf | Respiratory Distress Syndrome in Premature Infants (PIRDS) |
