# Supplementary material for: The availability and affordability of orphan drugs for rare diseases in China
Source: Orphanet J Rare Dis. 2016 Feb 27;11:20. doi: 10.1186/s13023-016-0392-4 (PMC4769558; doi:10.1186/s13023-016-0392-4)
Supplement: Additional file 2: Table S2. — The questionnaire on the availability and prices of orphan drugs for several serious rare diseases in hospitals. (DOC 248 kb) [file 13023_2016_392_MOESM2_ESM.doc]

**Additional file 2:** Table S2. The questionnaire on the availability and prices of orphan drugs for several serious rare diseases in hospitals

**Instruction:** In order to improve the quality of life and availability of orphan drugs of patients with rare diseases in China, we have designed this questionnaire to know the availability and prices of orphan drugs for several serious rare diseases in hospitals. We hope to get your support. Please fill in the following form carefully and put forward your suggestions for the survey. The information you provide will remain strictly confidential and will only be used for academic study. Thank you very much for your support and help.

Department of Pharmacy Business and Administration, School of Pharmacy, Tongji Medical College of Huazhong University of Science and Technology

2012.3.18

几种严重罕见疾病用药品在医疗机构的可获得性和价格调查表

为了改善我国严重罕见疾病患者的生命质量，促进严重疾病罕见疾病患者的用药可及性，我们特设计此调查表，以了解几种严重罕见疾病用药在医疗机构的可获得性和价格，希望贵医院能支持本次调查，真实填写调查表，并欢迎对此项研究提出宝贵意见和建议。我们保证调查所获得的数据仅用于科学研究，并保密单位和调查信息。对贵院的支持与帮助，我们在此表示衷心的感谢！

华中科技大学同济医学院药学院医药商业与管理系

2012.3.18

医院名称(Hospital Name)： 医院级别（Hospital Level）：

医院地址(Hospital Address)： 邮编号码(ZIP code)：

药师的姓名(Name of Pharmacist)： 手机号(Phone Number)：

| 药品通用名  Generic name | 产品名和商品名  Chinese product or brand name(s) | 剂型  Dosage  form | 规格  Strength  recommended | 是否有货  Available | | 药品调剂价格  Hospital dispensary price | | 制药企业  Manufacturer |
| --- | --- | --- | --- | --- | --- | --- | --- | --- |
|  |  |  |  | 有  yes | 无  no | 价格  Price of Pack Found | 规格  Pack Size Found |  |
| 白消安（Busulfan） | 白消安片 | 片剂(Tab) | 2mg |  |  |  |  |  |
|  |  |  | 0.5mg |  |  |  |  |  |
|  | 白消安片（马利兰） | 片剂(Tab) | 2mg |  |  |  |  |  |
|  | 白消安注射液（白舒非） | 注射剂(Inj) | 10ml：60mg |  |  |  |  |  |
| 高三尖杉酯碱（Homoharringtonine） | 高三尖杉酯碱注射液 | 注射剂(Inj) | 2ml:2mg |  |  |  |  |  |
|  |  |  | 1ml:1mg |  |  |  |  |  |
|  | 高三尖杉酯碱氯化钠注射液（川山宁、沃汀、扶尔） | 注射剂(Inj) | 250ml |  |  |  |  |  |
|  |  |  | 100ml |  |  |  |  |  |
|  | 注射用高三尖杉酯碱（赛兰） | 冻干粉针剂(Inj) | 2mg |  |  |  |  |  |
|  |  |  | 1mg |  |  |  |  |  |
| 米托蒽醌（Mitoxantrone Hydrochloride） | 注射用盐酸米托蒽醌（恒恩） | 注射剂(Inj) | 5mg |  |  |  |  |  |
|  |  | 注射剂  (冻干) (Inj) | 5mg |  |  |  |  |  |
|  | 盐酸米托蒽醌注射液（米西宁） | 注射剂(Inj) | 10ml:10mg |  |  |  |  |  |
|  |  |  | 2ml:2mg |  |  |  |  |  |
|  |  |  | 5ml:5mg |  |  |  |  |  |
|  | 盐酸米托蒽醌氯化钠注射液（泽康） | 注射剂(Inj) | 100ml:5mg |  |  |  |  |  |
| 甲异靛（Meisoindigo） | 甲异靛片 | 片剂(Tab) | 25mg |  |  |  |  |  |
| 甲磺酸伊马替尼（Imatinib Mesylate） | 甲磺酸伊马替尼片（格列卫） | 片剂(Tab) | 0.4g |  |  |  |  |  |
|  |  |  | 0.1g |  |  |  |  |  |
|  | 甲磺酸伊马替尼胶囊（格列卫） | 胶囊剂(Cap) | 0.1g |  |  |  |  |  |
| 达沙替尼（Dasatinib） | 达沙替尼片（施达赛） | 片剂(Tab) | 20mg |  |  |  |  |  |
|  |  |  | 20mg |  |  |  |  |  |
|  |  |  | 20mg |  |  |  |  |  |
|  |  |  | 20mg |  |  |  |  |  |
|  |  |  | 50mg |  |  |  |  |  |
|  |  |  | 50mg |  |  |  |  |  |
|  |  |  | 50mg |  |  |  |  |  |
|  |  |  | 50mg |  |  |  |  |  |
|  |  |  | 70mg |  |  |  |  |  |
|  |  |  | 70mg |  |  |  |  |  |
|  |  |  | 70mg |  |  |  |  |  |
|  |  |  | 70mg |  |  |  |  |  |
|  |  |  | 100mg |  |  |  |  |  |
|  |  |  | 100mg |  |  |  |  |  |
|  |  |  | 100mg |  |  |  |  |  |
|  |  |  | 100mg |  |  |  |  |  |
|  |  |  | 100mg |  |  |  |  |  |
| 尼洛替尼（Nilotinib） | 尼洛替尼胶囊(达希纳) | 胶囊剂(Cap) | 200mg |  |  |  |  |  |
|  |  |  |  |  |  |  |  |  |
| 替尼泊苷（Teniposide） | 替尼泊苷注射液（邦莱） | 注射剂(Inj) | 5ml:50mg |  |  |  |  |  |
|  | 替尼泊苷注射液（卫萌） | 注射剂(Inj) | 5ml:50mg |  |  |  |  |  |
| 腺嘌呤（Vitamin B4） | 磷酸腺嘌呤片（维生素B4片） | 片剂(Tab) | 10mg |  |  |  |  |  |
| 亚砷酸（Arsenious Acid and Sodium Chloride） | 亚砷酸氯化钠注射液（伊泰达） | 注射剂(Inj) | 5ml:5mg |  |  |  |  |  |
|  |  |  | 10ml:10mg |  |  |  |  |  |
| 利妥昔单抗（Rituximab） | 利妥昔单抗注射液（美罗华） | 注射剂(Inj) | 500mg/50ml |  |  |  |  |  |
|  |  |  | 100mg/10ml |  |  |  |  |  |
| 甲苯磺酸索拉非尼（Sorafenib Tosylate） | 甲苯磺酸索拉非尼片（多吉美） | 片剂(Tab) | 0.2g |  |  |  |  |  |
| 甲磺酸去铁胺（Desferrioxamine Mesilate） | 注射用甲磺酸去铁胺（得斯芬） | 注射剂(Inj) | 0.5g |  |  |  |  |  |
| 人凝血因子Ⅷ（Human Coagulation Factor Ⅷ） | 人凝血因子Ⅷ（康斯平） | 注射剂（无菌粉末）(Inj) | 400IU/10ml/瓶 |  |  |  |  |  |
|  |  |  | 100IU/10ml/瓶 |  |  |  |  |  |
|  |  |  | 200IU/10ml/瓶 |  |  |  |  |  |
|  |  |  | 50IU/10ml/瓶 |  |  |  |  |  |
|  | 人凝血因子Ⅷ | 注射剂(Inj) | 300IU(10ml)/瓶 |  |  |  |  |  |
|  |  | 冻干粉针剂(Inj) | 200 IU/瓶 |  |  |  |  |  |
|  |  |  | 100 IU/瓶 |  |  |  |  |  |
|  |  |  | 1000 IU/瓶 |  |  |  |  |  |
|  |  |  | 250 IU/瓶 |  |  |  |  |  |
|  |  |  | 300 IU/瓶 |  |  |  |  |  |
|  |  |  | 400 IU/瓶 |  |  |  |  |  |
|  |  |  | 500 IU/瓶 |  |  |  |  |  |
|  | 冻干人凝血因子Ⅷ | 冻干粉针剂(Inj) | 50 IU/瓶 |  |  |  |  |  |
|  |  |  | 200 IU/瓶 |  |  |  |  |  |
|  |  |  | 100 IU/瓶 |  |  |  |  |  |
| 重组人凝血因子Ⅷ（Recombinant Coagulation Factor VIII） | 注射用重组人凝血因子Ⅷ（拜科奇） | 粉针剂(Inj) | 1000IU/瓶 |  |  |  |  |  |
|  |  |  | 500IU/瓶 |  |  |  |  |  |
|  |  |  | 250IU/瓶 |  |  |  |  |  |
| 重组人凝血因子Ⅶa（Recombinant Human Coagulation Factor VIIa） | 注射用重组人凝血因子Ⅶa（诺其） | 注射剂(Inj) | 1mg(50KIU)/支 |  |  |  |  |  |
|  |  |  | 2mg(100KIU)/支 |  |  |  |  |  |
|  |  |  | 5mg(250KIU)/支 |  |  |  |  |  |
|  |  |  | 1.2mg（60KIU）/支 |  |  |  |  |  |
|  |  |  | 4.8mg（240KIU）/支 |  |  |  |  |  |
| 人凝血酶原复合物（Human Prothrombin Complex） | 人凝血酶原复合物（康舒宁) | 注射剂(无菌粉末) (Inj) | 1000IU/20ml/瓶 |  |  |  |  |  |
|  |  |  | 200IU/20ml/瓶 |  |  |  |  |  |
|  |  |  | 300IU/20ml/瓶 |  |  |  |  |  |
|  |  |  | 400IU/20ml/瓶 |  |  |  |  |  |
|  |  |  | 100IU/10ml/瓶 |  |  |  |  |  |
|  | 人凝血酶原复合物 | 注射剂(Inj) | Ⅸ,Ⅱ,Ⅹ3300IU/瓶 Ⅶ 200IU |  |  |  |  |  |
|  |  | 冻干粉针剂(Inj) | 300IU/瓶 |  |  |  |  |  |
|  |  |  | 1000IU/瓶 |  |  |  |  |  |
|  |  |  | 200IU/瓶 |  |  |  |  |  |
|  |  |  | 100IU/瓶 |  |  |  |  |  |
|  |  | 注射剂(Inj) | 300IU/瓶 |  |  |  |  |  |
|  |  |  | 400IU/瓶 |  |  |  |  |  |
|  |  |  | 200IU/瓶 |  |  |  |  |  |
|  | 冻干人凝血酶原复合物（康舒宁) | 冻干粉针剂(Inj) | 200 IU/瓶 |  |  |  |  |  |
|  |  |  | 100 IU/瓶 |  |  |  |  |  |
|  |  |  | 1000 IU/瓶 |  |  |  |  |  |
|  |  |  | 400 IU/瓶 |  |  |  |  |  |
|  |  |  | 300 IU/瓶 |  |  |  |  |  |
| 重组人生长激素（Somatropin,,Recombinant Human Growth Hormone） | 注射用重组人生长激素(安苏萌) | 注射剂(Inj) | 10IU/3.33mg/支 |  |  |  |  |  |
|  |  |  | 6IU/2mg/支 |  |  |  |  |  |
|  |  |  | 16IU/5.33mg/支 |  |  |  |  |  |
|  |  |  | 2IU/0.67mg/支；4.5IU/1.8mg/支 |  |  |  |  |  |
|  |  |  | 4.5IU/1.5mg/支 |  |  |  |  |  |
|  |  |  | 4IU/1.33mg/支 |  |  |  |  |  |
|  | 注射用重组人生长激素(珍怡） |  | 2.0mg(5IU) |  |  |  |  |  |
|  | 注射用重组人生长激素(赛高路） |  | 2.60mg/支(8IU/支) |  |  |  |  |  |
|  |  |  | 3.25mg/支(10IU/支) |  |  |  |  |  |
|  |  |  | 0.65mg/支(2IU/支) |  |  |  |  |  |
|  |  |  | 1.3mg(4IU/支) |  |  |  |  |  |
|  | 注射用重组人生长激素(思真） | 注射剂(Inj) | 8mg(24IU) |  |  |  |  |  |
|  |  |  | 4IU(1.33mg) |  |  |  |  |  |
|  |  |  | 10IU(3.33mg) |  |  |  |  |  |
|  | 注射用重组人生长激素(健豪宁） | 注射剂(Inj) | 5.3mg(16IU) |  |  |  |  |  |
|  | 注射用重组人生长激素(优猛茁） | 注射剂(Inj) | 5mg(15IU) |  |  |  |  |  |
|  | 注射用重组人生长激素(健豪宁） | 注射剂(无菌粉末) (Inj) | 5.3mg(16IU) |  |  |  |  |  |
|  | 注射用重组人生长激素(珍怡） | 冻干粉针剂(Inj) | 1.6mg(4IU) |  |  |  |  |  |
|  |  | 冻干制剂(Inj) | 1.2mg(3IU) |  |  |  |  |  |
|  |  |  | 1.0mg(2.5IU) |  |  |  |  |  |
|  |  |  | 4.0mg(10IU) |  |  |  |  |  |
|  | 注射用重组人生长激素(赛高路） |  | 2mg/支(6IU/支) |  |  |  |  |  |
|  | 重组人生长激素注射液(赛增) | 注射剂(Inj) | 60IU/20mg/3ml/瓶 |  |  |  |  |  |
|  |  |  | 1.5ml:15IU/5mg |  |  |  |  |  |
|  |  |  | 15IU/5mg/3ml/瓶 |  |  |  |  |  |
|  |  |  | 30IU/10mg/3ml/瓶 |  |  |  |  |  |
|  |  | 冻干粉针剂(Inj) | 2.5IU/0.85mg/1.0ml/瓶 |  |  |  |  |  |
|  |  |  | 4.0IU/1.33mg/1.0ml/瓶 |  |  |  |  |  |
|  |  |  | 4.5IU/1.7mg/1.0ml/瓶 |  |  |  |  |  |
|  |  |  | 10IU/3.7mg/1.0ml/瓶 |  |  |  |  |  |
| 伊米苷酶（Imiglucerase） | 注射用伊米苷酶（思而赞） | 注射剂(Inj) | 400单位/瓶 |  |  |  |  |  |
|  |  |  | 200单位/瓶 |  |  |  |  |  |
| 沙丙蝶呤（Sapropterin Dihydrochloride） | 盐酸沙丙蝶呤片（科望） | 片剂  (Tab) | 100mg |  |  |  |  |  |
|  |  |  | 100mg |  |  |  |  |  |
| 波生坦（Bosentan） | 波生坦片（全可利） | 片剂(Tab) | 125mg |  |  |  |  |  |
|  |  |  | 62.5mg |  |  |  |  |  |
| 伊洛前列素（Iloprost Solution） | 吸入用伊洛前列素溶液（万他维） | 溶液剂(Sol) | 2ml：20ug |  |  |  |  |  |
| 安立生坦（Ambrisentan） | 安立生坦片(凡瑞克) | 片剂(Tab) | 5mg |  |  |  |  |  |
|  |  |  | 10mg |  |  |  |  |  |
| 达那唑（Danazol） | 达那唑栓 | 栓剂(Sup) | 50mg |  |  |  |  |  |
|  | 达那唑胶囊 | 胶囊剂(Cap) | 0.2g |  |  |  |  |  |
|  |  |  | 0.2g |  |  |  |  |  |
|  |  |  | 0.1g |  |  |  |  |  |
|  |  |  | 1.5ml:0.12g |  |  |  |  |  |
| 猪肺磷脂注射液（Poractant Alfa） | 猪肺磷脂注射液(固尔苏) | 注射剂(Inj) | 3ml:0.24g |  |  |  |  |  |
|  |  |  | 1.5ml:0.12g |  |  |  |  |  |
| 利鲁唑（Riluzole） | 利鲁唑片（力如太） | 片剂(tab) | 50mg |  |  |  |  |  |
|  | 利鲁唑胶囊（协一力） | 胶囊剂(Cap) | 50mg |  |  |  |  |  |

Tab: tablet Cap: capsule Inj: injection Sol: solutions Sup: suppository

调查人(Investigator Name)： 问卷编号(Questionnaire Number)：

调查时间(Investigation time)：
