# Supplementary material for: The availability and affordability of orphan drugs for rare diseases in China
Source: Orphanet J Rare Dis. 2016 Feb 27;11:20. doi: 10.1186/s13023-016-0392-4 (PMC4769558; doi:10.1186/s13023-016-0392-4)
Supplement: Additional file 3: Table S3. — The unit prices of brand name and generic orphan drugs surveyed in 24 public tertiary hospitals in China. (DOC 53 kb) [file 13023_2016_392_MOESM3_ESM.doc]

**Additional file 3:**

Table S3. The unit prices of brand name and generic orphan drugs surveyed in 24 public tertiary hospitals in China

| **Generic name**  **(Brand name)** | **Dosage Form** | **Minimum Strength** | **Pack size** | **Minimum Strength Price (USD)** | **Median Unit Price (** **USD)** |
| --- | --- | --- | --- | --- | --- |
| **Originator Brands (22)** |  |  |  | **Median (Min-Max)** |  |
| Imiglucerase (Cerezyme) | Inj | 200IU | 1 vial | 817.3 * | 4.1/IU |
| Sapropterin dihydrochloride (Kuvan) | T | 100mg | 120 tablets | 47.1 * | 0.5/mg |
| Recombinant coagulation factor VIII (Kogenate FS) | Inj | 250IU | 1 kit | 211.5（177.6-211.5） | 0.8/ IU |
| Coagulation factor VIIa (Novoseven) | Inj | 1mg(50KIU) | 1 vial | 869.0（742.5-1061.1） | 869.0/mg |
| Bosentan (Tracleer) | T | 62.5mg | 56 tablets | 47.0（28.6-65.5） | 0.8/mg |
| Iloprost (Ventavis) | Inh | 2ml:20ug | 5 ampules | 87.5（80.5-87.5） | 4.4/ug |
| Ambrisentan (Volibris） | T | 5mg | 30 tablets | No data | -- |
| Somatropin (Saizen) | Inj | 4IU(1.33mg) | 1 kit | 43.6（36.9-60.2） | 10.9/ IU |
| Somatropin (Genotropin) | Inj | 5.3mg(16IU) | 5 vials | No data | -- |
| Somatropin (Humatrope) | Inj | 5mg(15IU) | 1 kit | 110.5 * | 7.4/mg |
| Busulfan (Busulfex ) | Inj | 10ml:60mg | 1 vial | 279.8（268.4-281.5） | 4.7/mg |
| Imatinib Mesylate (Glivec) | T | 0.1g | 60 tablets | 32.0（31.4-32.8） | 0.3/mg |
| Imatinib Mesylate (Glivec) | C | 0.1g | 120 capsules | 32.3（31.5-34.1） | 0.3/mg |
| Dasatinib (Sprycel) | T | 20mg | 14,28,56 or 60 tablets | No data | -- |
| Nilotinib (Tasigna) | C | 200mg | 28 or 112 capsules | 51.7（49.2-51.7） | 0.3/mg |
| Meisoindigo | T | 25mg | 50 tablets | No data | -- |
| Teniposide (Vumon) | Inj | 5ml:50mg | 10 vials | 28.0（25.2-29.0） | 0.6/mg |
| Arsenious acid (Yitaida) | Inj | 5ml:5mg | 2 vials | 11.6（11.1-13.7） | 2.3/mg |
| Rituximab (MabThera) | Inj | 10ml:100mg | 2 vials | 609.9（542.3-670.3） | 6.1/mg |
| Sorafenib (Nexavar) | T | 0.2g | 60 tablets | 67.1（62.4-71.5） | 0.3/mg |
| Riluzole (Rilutek) | T | 50mg | 56 tablets | No data | -- |
| Poractant alfa (Curosurf) | Inj | 1.5ml:0.12g | 1 vial | 562.0（509.1-627.4） | 4.7/mg |
| Generics (9) | Form | Strength | Pack size | -- | -- |
| Human coagulation factor Ⅷ | Inj | 200IU | 1 ampule | 60.9（44.5-80.1） | 0.3/IU |
| Human prothrombin complex concentrate | Inj | 200IU | 1 ampule | 30.6（25.4-34.6） | 0.2/ IU |
| Danazol | C | 0.1g | 30 capsules | 0.4（0.3-0.4） | 0.004/mg |
| Recombinant human growth hormone | Inj | 2IU | 1 kit | 7.4（6.0-19.3） | 3.7/ IU |
| Teniposide | Inj | 5ml:50mg | 5 vials | 21.9（19.6-27.4） | 0.4/ mg |
| Mitoxantrone hydrochloride | Inj | 5mg | 1 vial | 6.2（4.8-10.0） | 1.2/mg |
| Riluzole | T | 50mg | 56 tablets | 8.1（6.9-5.7） | 0.2/mg |
| Riluzole | C | 50mg | 24 capsules | 5.6（4.5-5.7） | 0.1/mg |
| Homoharringtonine | Inj | 1ml:1mg | 10 vials | 0.8（0.6-1.0） | 0.8/mg |

C: Capsules, T: Tablets, Inj: Injection, Inh: Inhalants. * Only one hospital dispensary price.
