# Supplementary material for: The availability and affordability of orphan drugs for rare diseases in China
Source: Orphanet J Rare Dis. 2016 Feb 27;11:20. doi: 10.1186/s13023-016-0392-4 (PMC4769558; doi:10.1186/s13023-016-0392-4)
Supplement: Additional file 4: Table S4. — The daily dose and course of treatment for the surveyed orphan drugs. (DOC 52 kb) [file 13023_2016_392_MOESM4_ESM.doc]

**Additional file 4: Table S4. The daily dose and course of treatment for the surveyed orphan drugs**

| Generic name (Brand name) | Indicated disease | Dosage | Daily Dose | Duration days of a treatment course |
| --- | --- | --- | --- | --- |
| Human coagulation  fcctor Ⅷ | HEM | The required amount of F Ⅷ (IU) / time = 0.5 × patient weight (kg) × the need to elevate the activity level of F Ⅷ (the percentage of the normal %)  The generally recommended dose is as follows:  1. Mild to moderate bleeding: a single dose of 10 ~ 15 IU / kg, increase the level of factor Ⅷ to 20%- 30% of normal;  2. More serious bleeding or minor surgery: increase factor Ⅷ to 30 %-50% of the normal level, usually the first dose is 15 ~ 25 IU / kg. Every 8 to 12 hours, if necessary, give the maintenance dose of 10 ~ 15 IU / kg;  3. Life-threatening bleeding: the first dose is 40 IU / kg, and then every 8 to 12 hours to give a maintenance dose of 20 ~ 25 IU / kg. The duration of treatment is determined by doctors;  4. Surgery: at the beginning of surgery, the blood concentration of factor Ⅷ needs reach to the 60%-120% of the normal levels. Usually, in the pre operative 30 ~ 40 IU / kg administered. 4 days after the surgery, the minimum of level factor Ⅷ should be maintained at the 60%of normal level; In the next four days, it can be reduced to 40%. | 1750IU | 2 |
| Recombinant human coagulation fcctor VIII  (Kogenate FS) | HEM | Dose (IU) = weight (kg) * the percentage expected increase value of F Ⅷ % / 2% / IU / kg. For hemophilic patients, when increased to 10% -20% of normal levels, factor VIII can help to maintain the hemostatic effect of spontaneous bleeding; At 20% -30% of normal levels, factor VIII can be used for hemophilic patients who require tooth extraction and such minor surgery or have been involved in minor trauma; when serum levels increase to more than 50% of normal levels, these patients can undergo surgery. The half-life of factor VIII is 10-12 hours, and should be infused twice a day. | 1750IU | 2 |
| Recombinant human coagulation fcctor VIIa  (NovoSeven) | HEM | 1.Mild to moderate bleeding situations (including out-patient treatment)  For treatment of outpatients, the dose is set at 90ug / kg, which can be effective in the treatment of mild to moderate joint, muscle, mucous membrane and skin bleeding. Given at 2-3 hour intervals. For outpatient care, the course of treatment should not exceed 24 hours.  2. Patients with severe bleeding or major surgery can take this drug for to 2-3 weeks. | 50.4mg | 1 |
| Human prothrombin complex concentrate | HEM | 1. The dose differs with the degree of coagulation factor deficiency, but generally from 10 to 20 units per kilogram. The lack of coagulation factor VII requires infusion every 6 to 8 hours, every 24 hours for coagulation factor IX deficiency, and every 24 to 48 hours for coagulation factor II and coagulation factorⅹ deficiency. The treatment usually lasts for 2 to 3 days.  2. For large amount of bleeding or major surgery, the dosage can be increased according to the patient’s condition. | 3150IU | 2 |
| Bosentan | PAH | Maintenance dose: 125 mg once time, twice a day.  DDD of WHO is 250mg per day. | 250mg | 28 |
| Iloprost  (Ventavis) | PAH | Inhalation 6-9 times a day，single dose is 2.5ug-5ug.  DDD of WHO is 50ug per day. | 50ug | 28 |
| Recombinant human growth hormone | GHD | Recommended daily dose is 0.1 IU/kg | 7IU | 28 |
| Busulfan （Busulfex） | CML | Adult dose is usually 0.8mg/kg, administered once every 6 hours, 4 consecutive days (a total of 16times). | 224mg | 4 |
| Imatinib (Glivec） | CML | The recommended dose for blasted phase accelerated phase patients is 600mg /day; chronic phase is 400mg/day. As long as effective, they should continue taking. | 600mg | 28 |
| Nilotinib (Tasigna) | CML | For patients with Ph ^+^ CML in chronic phase or accelerated phase. Dosing is 400mg, twice daily. As long as the patients derive benefit, the treatment should be continued. | 800mg | 28 |
| Teniposide (Vumon) | ALL | 60mg/m^2^，once a day，continue 5 days，interval of 3 weeks. | 102mg | 5 |
| Mitoxantrone | AML | According to body surface area one dose 12 ~ 14mg/m^2^, once every 3 to 4 weeks; Or according to body surface area, 8mg/m^2^, once a day, once every three to five days, interval of 2 to 3 weeks. | 10.2mg | 4 |
| Homoharringtonine | AML | Daily 1 ~ 4mg, continuous infusion over 40 to 60 days, or 4 to 6 days for a course, intermittent 1to 2 weeks, reuse. | 2.5mg | 5 |
| Arsenious acid | APL | Adult: once a day, each time 10mg or 7mg/m^2^, 4 weeks as a course. | 10mg | 28 |
| Rituximab （MabThera） | NHL | 375 mg/m^2^, once a week, total 4 times. | 91.1mg | 28 |
| Sorafenib tosylate  (Nexavar) | RCC | Initially 0.4g (2×0.2g), twice a day. Continue treatment until benefit disappears or toxicity is intolerable. | 800mg | 28 |
| Danazol | HAE | Maintenance dose is 200mg a day, 2 or 3 times a day until the efficacy appears.  DDD of WHO is 600mg per day. | 600mg | 28 |
| Riluzole (Rilutek) | ALS | Single dose 50mg, twice day.  DDD of WHO is 100mg per day. | 100mg | 28 |
| Poractant alfa (Curosurf) | PIRDS | The recommended dose is 100-200mg/kg (1.25-2.5mL/kg), every 12 hours and then 100mg extra as needed (maximum total dose: 300-400 mg/kg). | 525mg | 1 |

1. In the calculation of hemophilia, we choose mild to moderate bleeding situation and increase the plasma level of factor Ⅷ to 25% of normal. 2. In the calculations, we used the following average values: adult weight at 70kg, children 15kg, baby 1.5kg; the body surface area at 1.7m^2^. 3. Recombinant human coagulation factor VIIa (NovoSeven): Recommended dose is 90ug/kg, 8 times a day. 4. Poractant alfa (Curosurf): Birth weight used was 1.5kg. The first dose is 150mg/kg; repeat dose is 100mg/kg, only one day usage.
