# Supplementary material for: The availability and affordability of orphan drugs for rare diseases in China
Source: Orphanet J Rare Dis. 2016 Feb 27;11:20. doi: 10.1186/s13023-016-0392-4 (PMC4769558; doi:10.1186/s13023-016-0392-4)
Supplement: Additional file 5: Table S5. — List and general situation of 165 unique orphan drugs marketed in China. (DOC 316 kb) [file 13023_2016_392_MOESM5_ESM.doc]

**Additional file 5:** Table S5. List and general situation of 165 unique orphan drugs marketed in China

| ATC code | Generic name | Brand name | Chinese drug name | Manufacture or import status | Strength | Per Strength Price | Average Unit Price of drug Procurement (USD) * | Number of procurement provinces | EM |
| --- | --- | --- | --- | --- | --- | --- | --- | --- | --- |
|  |  |  |  |  |  | Average(Min-Max)  (USD) |  |  | Y/N |
| A01AB18 | Clotrimazole | Empecid | 克霉唑乳膏 | M and I | 10g:300mg | 0.13（0.08，0.22） | 0.0004/mg | 11 | N |
| A03AB02 | Glycopyrrolate | Cuvposa | 格隆溴铵片 | M | 0 | 0 | 0 | 0 | N |
| A05AA02 | Ursodeoxycholic Acid | URSO | 熊去氧胆酸片 | M and I | 50mg | 0.03（0.001，0.04） | 0.0007/mg | 11 | Y |
| A07AA11 | Rifaximin | Normix | 利福昔明片 | M and I | 200mg | 0.48（0.29，1.68） | 0.002/mg | 5 | N |
| A07EC02 | Mesalazine | PENTASA | 美沙拉嗪肠溶片 | M | 25mg | 0.24（0.24，0.24） | 0.001/mg | 4 | N |
| A12AA12 | Calcium acetate | Phos-Lo | 醋酸钙片 | M | 66.7mg | 10.39（8.23，13.30） | 0.001/mg | 4 | N |
| A16AA01 | Levocarnitine | Carnitor | 左卡尼汀注射液 | M and I | 5ml:1000mg | 5.63（1.59，9.49） | 0.006/mg | 5 | N |
| A16AA03 | Glutamine (L) | Nutrestore | L谷氨酰胺呱仑酸钠颗粒 | M and I | 100ml:20000mg | 21.01（13.50，24.36） | 0.001/mg | 8 | N |
| A16AB02 | Imiglucerase | Cerezyme | 注射用伊米苷酶 | I | 0 | 0 | 0 | 0 | N |
| A16AX07 | Sapropterin dihydrochloride | Kuvan | 盐酸沙丙蝶呤片 | I | 0 | 0 | 0 | 0 | N |
| B01AC11 | Iloprost inhalation solution | Ventavis | 吸入用伊洛前列素溶液 | I | 0 | 0 | 0 | 0 | N |
| B01AC19 | Beraprost Sodium | DORNER | 贝前列素钠片 | I | 20ug | 0.79（0.78，0.82） | 0.04/mg | 5 | N |
| B01AE03 | Argatroban Hydrate | Novastan | 阿加曲班注射液 | M and I | 20ml:10mg | 23.01（11.88，25.58） | 2.30/mg | 5 | N |
| B02AB01 | Aprotinin | Trasylol | 注射用抑肽酶 | M | 0 | 0 | 0 | 0 | N |
| B02BD04 | Coagulation Factor IX (recombinant) | Benefix | 注射用重组人凝血因子IX | I | 0 | 0 | 0 | 0 | N |
| B02BD05 | Coagulation Factor VIIa (Recombinant) | Novoseven | 注射用重组人凝血因子VIIa | I | 1.2mg(60KU) | 1057.57（1057.45，1057.69） | 881.31/mg | 3 | N |
| B02BD08 | Eptacog Alfa (Activated)(Genetical Recombination) | NovoSeven | 注射用重组人凝血因子VIIa | I | 0 | 0 | 0 | 0 | N |
| B02BD09 | Nonacog Alfa (Genetical Recombination) | BeneFIX | 注射用重组人凝血因子IX | I | 0 | 0 | 0 | 0 | N |
| B03XA01 | Epoetin alfa | Epogen | 重组人促红素注射液(CHO细胞） | M and I | 1ml:1000IU | 1.67（1.14，18.59） | 0.0027/ IU | 8 | N |
| C01BD01 | Amiodarone HCl | Cordarone | 盐酸胺碘酮片 | M and I | 200mg | 0.13（0.003，0.40） | 0.0007/mg | 15 | Y |
| C01CA17 | Midodrine HCl | ProAmatine | 盐酸米多君片 | M and I | 2.5mg | 0.38（0.32，0.43） | 0.15/mg | 3 | N |
| C02KX01 | Bosentan | Tracleer | 波生坦片 | I | 125mg | 56.96（56.96，56.96） | 0.46/mg | 1 | N |
| C02KX02 | Ambrisentan | Letairis | 安立生坦片 | I | 5mg | 51.13（51.13，51.13） | 10.22/mg | 1 | N |
| C07AA07 | Sotalol HCl | Betapace | 盐酸索他洛尔片 | M | 80mg | 0.18（0.19，0.21） | 0.002/mg | 5 | N |
| C09AA16 | Imidapril Hydrochloride | Tanatril | 盐酸咪达普利片 | M | 5mg | 0.26（0.25，0.27） | 0.05/mg | 2 | N |
| D06BX01 | Metronidazole | Metrogel | 甲硝唑乳膏 | M and I | 300mg:10000mg | 0.68（0.68，0.68） | 0.002/mg | 1 | N |
| G03AC05 | Megestrol acetate | Megace | 醋酸甲地孕酮分散片 | M | 160mg | 1.55（1.46，1.64） | 0.01/mg | 5 | N |
| G03DA03 | Hydroxyprogesterone caproate | Makena | 复方已酸羟孕酮注射液 | M | 0 | 0.00 | 0 | 0 | N |
| G03GA04 | Urofollitropin | Metrodin | 注射用尿促卵泡素 | M | 75IU | 22.70（21.88，23.52） | 0.30/IU | 3 | N |
| G03GA05 | Follitropin alfa, recombinant | Gonal-F | 重组人促卵泡激素注射液 | I | 100IU | 266.71（55.87，66.05） | 2.67/IU | 5 | N |
| G03XB01 | Mifepristone | Korlym | 米非司酮片 | M | 25mg | 0.56（0.53，0.61） | 0.02/mg | 9 | Y |
| G03XC01 | Raloxifene | Evista | 盐酸雷洛昔芬片 | M and I | 60mg | 1.39（1.09，1.84） | 0.02/mg | 4 | N |
| G04BE08 | Tadalafil | Adcirca | 他达拉非片 | I | 20mg | 17.85（17.06，18.11） | 0.89/mg | 4 | N |
| H01AC01 | Somatropin (rDNA origin) | Saizen | 注射用重组人生长激素（思真） | M and I | 10IU | 52.88（22.05，977.24） | 5.29/IU | 10 | N |
| H01AC01 | Somatropin | Genotropin | 注射用重组人生长激素（健豪） | M and I | 0 | 0 | 0 | 0 | N |
| H01AC01 | Somatropin | Norditropin，Norditropin SimpleXx | 聚乙二醇重组人生长激素注射液 （诺泽） | M and I | 30IU | 182.93（181.97，184.13） | 6.10/IU | 7 | N |
| H01BA02 | Desmopressin acetate | n/a | 醋酸去氨加压素注射液 | M and I | 1ml:1ug | 0.90（0.70，1.09） | 0.90/ug | 6 | Y |
| H01CA01 | Gonadorelin acetate | Lutrepulse | 注射用戈那瑞林 | M | 0.1mg | 2.03（1.92，2.25） | 20.29/mg | 4 | N |
| H01CB02 | Octreotide | Sandostatin Lar | 醋酸奥曲肽注射液 | M and I | 1ml:0.1mg | 8.52（1.46，19.08） | 85.20/mg | 6 | N |
| H01CB03 | Lanreotide | Somatuline Depot | 注射用醋酸兰瑞肽 | I | 40mg | 550.97（510.21，577.62） | 13.77/mg | 4 | N |
| H02AB09 | Hydrocortisone | Plenadren | 氢化可的松注射液 | M | 2ml:10mg | 0.03（0.02，0.06） | 0.003/mg | 12 | Y |
| H05AA02 | Teriparatide | Parathar | 特立帕肽注射液 | I | 2.4ml:20μg:80μl | 1106.25（1106.25，1106.25） | 55.31/ug | 1 | N |
| J01EC02 | Sulfadiazine | n/a | 磺胺嘧啶片 | M | 500mg | 0.01（0.01，0.02） | 0.00003/mg | 6 | Y |
| J01FA09 | Clarithromycin | Clarith | 克拉霉素胶囊 | M | 125mg | 0.13（0.10，0.32） | 0.001/mg | 5 | Y |
| J01FA10 | Azithromycin Hydrate | Zithromac | 阿奇霉素片 | M and I | 250mg | 0.36（0.04，1.70） | 0.001/mg | 13 | Y |
| J01MA01 | Ofloxacin | Tarivid | 氧氟沙星片 | M | 100mg | 0.04（0.01，0.25） | 0.0004/mg | 4 | Y |
| J01XA01 | Vancomycin Hydrochloride | Vancomycin | 注射用盐酸万古霉素 | M | 500mg | 16.10（13.69，22.30） | 0.03/mg | 7 | N |
| J02AA01 | Amphotericin B lipid complex | Abelcet | 注射用两性霉素B脂质体 | M and I | 10mg | 22.23（22.16，22.42） | 2.22/mg | 7 | N |
| J02AA01 | Liposomal amphotericin B | Ambisome | 注射用两性霉素B脂质体 | M and I | 10mg | 22.23（22.16，22.42） | 2.22/mg | 7 | N |
| J04AB02 | Rifampin(Rifampicin) | Rifadin I.V. | 利福平注射液 | M | 5ml:300mg | 4.46（4.15，4.96） | 0.015/mg | 6 | N |
| J04AM05 | Rifampin, isoniazid, pyrazinamide | Rifater | 异福酰胺片 | M | 450mg | 0.11（0.06，0.22） | 0.0002/mg | 3 | N |
| J04AB04 | Rifabutin | Mycobutin | 利福布汀胶囊 | M | 150mg | 2.56（2.41，2.64） | 0.017/mg | 5 | N |
| J04AB05 | Rifapentine | Priftin | 利福喷丁胶囊 | M | 150mg | 0.14（0.11，0.17） | 0.0009/mg | 7 | N |
| J04BA01 | Clofazimine | Lamprene | 氯法齐明软胶囊 | M | 50mg | 2.23（2.23，2.23） | 0.04/mg | 1 | N |
| J05AB04 | Ribavirin | Rebetol | 利巴韦林片 | I | 100mg | 0.02（0.0007，0.05） | 0.0002/mg | 11 | N |
| J05AB14 | Valganciclovir Hydrochloride | Valixa | 盐酸缬更昔洛韦片 | I | 450mg | 40.50（38.05，42.95） | 0.09/mg | 2 | N |
| J05AD01 | Foscarnet Sodium Hydrate | Foscavir | 膦甲酸钠氯化钠注射液 | M | 250ml:300mg | 15.78（12.50，21.47） | 0.005/mg | 5 | N |
| J05AE01 | Saquinavir Mesilate | Invirase | 甲磺酸沙奎那韦片 | M | 0 | 0 | 0 | 0 | N |
| J05AE02 | Indinavir Sulfate Ethanolate | Crixivan | 硫酸茚地那韦胶囊 | M | 0 | 0 | 0 | 0 | N |
| J05AE03 | Ritonavir | Norvir | 利托那韦片 | I | 0 | 0 | 0 | 0 | N |
| J05AE08 | Atazanavir Sulfate | REYATAZ | 硫酸阿扎那韦胶囊 | I | 0 | 0 | 0 | 0 | N |
| J05AF01 | Zidovudine | Retrovir | 齐多夫定注射液 | M | 0 | 0 | 0 | 0 | N |
| J05AF02 | Didanosine | Videx EC | 去羟肌苷肠溶胶囊 | M and I | 0 | 0 | 0 | 0 | N |
| J05AF06 | Abacavir Sulfate | Ziagen | 硫酸阿巴卡韦片 | I | 0 | 0 | 0 | 0 | N |
| J05AF07 | Tenofovir | Viread | 替诺福韦二吡呋酯片 | M and I | 0 | 0 | 0 | 0 | N |
| J05AF09 | Emtricitabine | Emtriva | 恩曲他滨片 | M and I | 0 | 0 | 0 | 0 | N |
| J05AG01 | Nevirapine | Viramune | 奈韦拉平片 | M and I | 0 | 0 | 0 | 0 | N |
| J05AG03 | Efavirenz | Stocrin | 依非韦伦片 | M and I | 0 | 0 | 0 | 0 | N |
| J05AG04 | Etravirine | Intelence | 依曲韦林片 | M | 0 | 0 | 0 | 0 | N |
| J05AG05 | Rilpivirine Hydrochloride | EDURANT | 利匹韦林片 | I | 0 | 0 | 0 | 0 | N |
| J05AR10 | Lopinavir, Ritonavir | Kaletra | 洛匹那韦利托那韦片 | I | 0 | 0 | 0 | 0 | N |
| J05AX08 | Raltegravir Potassium | ISENTRESS | 拉替拉韦钾片 | I | 0 | 0 | 0 | 0 | N |
| J05AX09 | Maraviroc | Celsentri | 马拉韦罗片 | I | 0 | 0 | 0 | 0 | N |
| J06BB04 | Hepatitis B immune globulin (human) | Hepagam | 乙型肝炎人免疫球蛋白 | M | 200IU | 47.76（35.77，52.88） | 0.24/IU | 2 | N |
| L01AA01 | Cyclophosphamide Hydrate | Endoxan | 注射用环磷酰胺 | M and I | 200mg | 2.08（0.55，4.53） | 0.01/mg | 9 | Y |
| L01AA03 | Melphalan | Alkeran For Injection | 美法仑片 | I | 0 | 0 | 0 | 0 | N |
| L01AA06 | Ifosfamide | Ifex | 注射用异环磷酰胺 | M and I | 1mg | 21.02（8.57，42.92） | 21.02/mg | 10 | N |
| L01AB01 | Busulfan | Busulfex | 白消安注射液 | I | 10ml:60mg | 269.39（269.39，269.39） | 4.49/mg | 1 | N |
| L01BA01 | Methotrexate sodium | Methotrexate | 甲氨蝶呤注射液 | M and I | 10ml:1000mg | 2.91（2.52，3.29） | 0.003/mg | 5 | Y |
| L01BA04 | Pemetrexed disodium | Alimta | 注射用培美曲塞二钠 | M and I | 500mg | 647.98（549.20，821.79） | 1.30/mg | 8 | N |
| L01BB02 | 6-mercaptopurine monohydrate | Xaluprine | 巯嘌呤片 | M | 50mg | 0.07（0.07，0.07） | 0.001/mg | 1 | Y |
| L01BB04 | Cladribine | Leustatin | 克拉屈滨注射液 | M | 10ml:10mg | 1068.43（1068.11，1068.75） | 106.84/mg | 3 | N |
| L01BB05 | Fludarabine phosphate | Fludara | 注射用磷酸氟达拉滨 | M | 50mg | 218.49（174.66，367.31） | 4.37/mg | 5 | N |
| L01BB05 | Fludarabine phosphate oral tablets | n/a | 磷酸氟达拉滨片 | I | 10mg | 71.03（71.03，71.03） | 7.10/mg | 1 | N |
| L01BC01 | Cytarabine | Cylocide N | 注射用盐酸阿糖胞苷 | M and I | 100mg | 2.92（1.20，8.18） | 0.03/mg | 6 | Y |
| L01BC05 | Gemcitabine Hydrochloride | Gemzar | 注射用盐酸吉西他滨 | M and I | 200mg | 55.20（27.19，72.79） | 0.28/mg | 5 | N |
| L01BC08 | Decitabine | Dacogen | 注射用地西他滨 | M and I | 50mg | 1667.47（1662.74，1670.51） | 33.35/mg | 6 | N |
| L01CB02 | Teniposide | Vumon | 替尼泊苷注射液 | M and I | 5ml:50mg | 21.83（18.99，25.14） | 0.44/mg | 7 | N |
| L01CD01 | Paclitaxel | Taxol | 紫杉醇注射液 | M and I | 5ml:30mg | 30.87（18.75，59.59） | 1.03/mg | 3 | Y |
| L01DB01 | Doxorubicin liposome | Doxil | 盐酸多柔比星脂质体注射液 | M and I | 10ml:20mg | 1058.65（836.22，1245.83） | 52.93/mg | 4 | N |
| L01DB03 | Epirubicin | Ellence | 注射用盐酸表柔比星 | M and I | 5ml:10mg | 47.57（15.39，108.88） | 4.76/mg | 10 | N |
| L01DB06 | Idarubicin HCl for injection | Idamycin | 注射用盐酸伊达比星 | M and I | 10mg | 475.91（381.19，623.08） | 47.59/mg | 5 | N |
| L01DB07 | Mitoxantrone | Novantrone | 注射用盐酸米托蒽醌 | M | 5mg | 6.02（5.37，7.53） | 1.20/mg | 5 | N |
| L01DC03 | Mitomycin-C | Mitosol | 注射用丝裂霉素 | M | 10mg | 11.45（6.47，12.92） | 1.14/mg | 7 | Y |
| L01XC02 | Rituximab | Rituxan | 利妥昔单抗注射液 | I | 50ml:500mg | 2892.55（2884.62，2900.48） | 5.79/mg | 6 | N |
| L01XC03 | Trastuzumab | Herceptin | 注射用曲妥珠单抗 | I | 440mg | 3638.46（3604.62，3660.32） | 8.27/mg | 6 | N |
| L01XC06 | Cetuximab | Erbitux | 西妥昔单抗注射液 | I | 20ml:100mg | 730.77（692.31，740.38） | 7.31/mg | 8 | N |
| L01XC07 | Bevacizumab | Avastin | 贝伐珠单抗注射液 | I | 4ml:100mg | 843.40（843.40，843.40） | 8.43/mg | 1 | N |
| L01XE01 | Imatinib | Gleevec | 甲磺酸伊马替尼片 | M and I | 100mg | 29.45(31.25,33.65) | 0.29/mg | 7 | N |
| L01XE04 | Sunitinib Malate | Sutent | 苹果酸舒尼替尼胶囊 | I | 12.5mg | 73.16（70.89，74.11） | 5.85/mg | 7 | N |
| L01XE05 | Sorafenib Tosylate | Nexavar | 甲苯磺酸索拉非尼片 | I | 200mg | 65.70（62.18，67.08） | 0.33/mg | 5 | N |
| L01XE06 | Dasatinib | Sprycel | 达沙替尼片 | M and I | 0 | 0 | 0 | 0 | N |
| L01XE08 | Nilotinib | Tasigna | 尼洛替尼胶囊 | I | 200mg | 53.17（52.08，54.29） | 0.27/mg | 5 | N |
| L01XE10 | Everolimus | Afinitor | 依维莫司片 | I | 0 | 0 | 0 | 0 | N |
| L01XE16 | Crizotinib | Xalkori | 克唑替尼胶囊 | I | 0 | 0 | 0 | 0 | N |
| L01XX03 | Altretamine | Hexalen | 六甲蜜胺胶囊 | M | 50mg | 0.88（0.88，1.05） | 0.02/mg | 8 | N |
| L01XX14 | Tretinoin | Vesanoid | 维A酸胶囊 | M | 0 | 0 | 0 | 0 | N |
| L01XX24 | Pegaspargase | Oncaspar | 培门冬酶注射液 | M | 5ml:3751IU | 727.36（691.29，772.44） | 0.2/IU | 7 | N |
| L01XX27 | Arsenic trioxide | Trisenox | 亚砷酸氯化钠注射液，注射用三氧化二砷 | M | 5ml:5mg | 37.90（37.90，37.90） | 7.59/mg | 1 | Y |
| L01XX32 | Bortezomib | Velcade | 注射用硼替佐米 | I | 1mg | 809.15（792.81，825.48） | 809.15/mg | 9 | N |
| L02BA02 | Toremifene | Fareston | 枸橼酸托瑞米芬片 | M and I | 60mg | 1.75（1.74，1.75） | 0.032/mg | 6 | N |
| L02BG06 | Exemestane | Aromasin | 依西美坦片 | M and I | 25mg | 5.20（1.69，8.59） | 0.21/mg | 7 | N |
| L03AB03 | Interferon Gamma-1a(Genetical Recombination) | Imunomax®-γ | 注射用重组人干扰素γ | I | 2Mill IU | 8.14（7.44，8.94） | 0.041/IU | 7 | N |
| L03AB04 | Interferon alfa-2a (recombinant) | Roferon-A | 注射用重组人干扰素α2a | M and I | 6Mill IU | 7.69（5.77，9.71） | 0.013/IU | 3 | N |
| L03AB05 | Interferon alfa-2b (recombinant) | Intron A | 注射用重组人干扰素α2b | M and I | 5Mill IU | 8.57（5.77，11.54） | 0.017/IU | 8 | N |
| L03AB08 | Interferon beta-1b | Betaseron | 注射用重组人干扰素β-1b | I | 0.3mg | 131.89（131.89，131.89） | 439.64/mg | 1 | N |
| L03AB10 | Peginterferon alfa-2b | Sylatron | 聚乙二醇干扰素α-2b注射剂 | I | 100ug | 212.58（210.38，214.76） | 2.13/ug | 2 | N |
| L03AX03 | Freeze-dried BCG Vaccine(Japanese Strain) | Immunobladder | 皮内注射用卡介苗 | I | 0 | 0 | 0 | 0 | N |
| L04 | Anti-human T-Lymphocyte Immunoglobulin, Rabbit | Zetbulin | 抗人T细胞兔免疫球蛋白 | M and I | 5ml:100mg | 604.65（604.65，604.65） | 6.05/mg | 1 | N |
| L04 | Mycophenolate Mofetil | CellCept | 吗替麦考酚酯分散片 | M and I | 250mg | 1.23（1.02，1.48） | 0.005/mg | 5 | N |
| L04AA04 | Anti-human Thymocyte Immunoglobulin, Rabbit | Thymoglobuline | 兔抗人胸腺细胞免疫球蛋白 | I | 5ml:25mg | 462.06（462.00，462.00） | 18.48/mg | 1 | N |
| L04AB01 | Etanercept | Enbrel | 注射用依那西普 | I | 25mg | 378.93（375.64，382.21） | 15.16/mg | 5 | N |
| L04AB02 | Infliximab | Remicade | 注射用英夫利西单抗 | I | 100mg | 981.64（938.78，1025.64） | 9.82/mg | 5 | N |
| L04AB04 | Adalimumab | Humira | 阿达木单抗注射液 | I | 0 | 0 | 0 | 0 | N |
| L04AC02 | Basiliximab (GeneticalRecombination) | Simulect | 注射用巴利昔单抗 | I | 20mg | 1356.86（1345.35，1368.36） | 67.84/mg | 5 | N |
| L04AD01 | Ciclosporin | Sandimmun | 环孢素注射液 | M and I | 5ml:250mg | 22.96（15.72，26.89） | 0.09/mg | 5 | N |
| L04AD02 | Tacrolimus | Prograf | 他克莫司胶囊 | M and I | 0.5mg | 1.91（1.57，2.23） | 3.82/mg | 6 | N |
| L04AX02 | Thalidomide | Thalomid | 沙利度胺胶囊 | M | 25mg | 0.36（0.35，0.37） | 0.01/mg | 1 | N |
| L04AX04 | Lenalidomide | Revlimid | 来那度胺胶囊 | I | 0 | 0 | 0 | 0 | N |
| M01AC06 | Meloxicam | Mobic | 美洛昔康片 | M and I | 7.5mg | 0.23（0.04，0.45） | 0.03/mg | 7 | N |
| M03AX01 | Botulinum toxin type A | Botox | 注射用A型肉毒毒素 | I | 0 | 0 | 0 | 0 | N |
| M03BX01 | Baclofen | Lioresal Intrathecal | 巴氯芬片 | M and I | 10mg | 0.27（0.21，0.49） | 0.03/mg | 7 | N |
| M04AC01 | Colchicine Tablets | Colcrys | 秋水仙碱片 | M and I | 10mg | 1.19（0.60，3.54） | 0.12/mg | 18 | Y |
| M05 | Etidronate disodium | Didronel | 依替膦酸二钠片 | M | 200mg | 0.33（0.24，0.42） | 0.002/mg | 5 | N |
| M05BA07 | Sodium Risedronate Hydrate | Actonel,Benet | 利塞膦酸钠片 | M | 5mg | 0.86（0.67，1.35） | 0.17/mg | 6 | N |
| N02CX02 | Clonidine | Duraclon | 盐酸可乐定注射液 | M | 0 | 0 | 0 | 0 | N |
| N03AX09 | Lamotrigine | Lamictal | 拉莫三嗪片 | M and I | 25mg | 0.26（0.18，0.34） | 0.01/mg | 8 | N |
| N03AX11 | Topiramate | Topamax | 托吡酯片 | M and I | 25mg | 0.26（0.22，0.30） | 0.01/mg | 6 | N |
| N03AX12 | Gabapentin | Gralise | 加巴喷丁片 | M and I | 100mg | 0.11（0.08，0.16） | 0.001/mg | 7 | N |
| N04BC07 | Apomorphine HCl | Apokyn | 盐酸阿扑吗啡注射液 | M | 0 | 0 | 0 | 0 | N |
| N04BD01 | Selegiline HCl | Eldepryl | 盐酸司来吉兰片 | M and I | 5mg | 0.51（0.48，0.53） | 0.10/mg | 3 | N |
| N05CA24 | Phenobarbital Sodium | Nobelbar | 苯巴比妥注射液 | M | 1ml:100mg | 0.07（0.05，0.10） | 0.0008/mg | 4 | Y |
| N06BC01 | Caffeine Citrate | Cafcit | 枸橼酸咖啡因注射液 | I | 0 | 0 | 0 | 0 | N |
| N07BC01 | Buprenorphine hydrochloride | Subutex | 盐酸丁丙诺啡舌下片 | M | 0 | 0 | 0 | 0 | N |
| N07XX02 | Riluzole | Rilutek | 利鲁唑片 | M and I | 50mg | 5.63（4.01，5.52） | 0.11/mg | 8 | N |
| P01AB02 | Tinidazole | Tindamax | 替硝唑片 | M | 500mg | 0.03（0.01，0.05） | 0.00007/mg | 13 | Y |
| P01BC01 | Quinine Sulfate | n/a | 硫酸奎宁片 | M | 0 | 0 | 0 | 0 | N |
| P01BF01 | Artemether 20mg /lumefantrine 120mg | Coartem | 复方蒿甲醚片 | M | 80mg | 1.28（1.28，1.28） | 0.02/mg | 2 | N |
| P02CA03 | Albendazole | Albenza | 阿苯达唑片 | M | 100mg | 0.02（0.0014，0.05） | 0.0002/mg | 12 | Y |
| P02CF01 | Ivermectin | Stromectol | 伊维菌素片 | M | 0 | 0 | 0 | 0 | N |
| R05CB12 | Tiopronin | Thiola | 硫普罗宁注射液 | M | 2ml:100mg | 1.74（1.89，2.80） | 0.02/mg | 5 | N |
| R07 | Poractant Alfa | Curosurf | 猪肺磷脂注射液 | M and I | 3ml:240mg | 1050.63（1032.45，1065.71） | 4.38/mg | 8 | N |
| S | Tacrolimus Hydrate | Talymus | 他克莫司滴眼液 | I | 0 | 0 | 0 | 0 | N |
| S01AD09 | Ganciclovir Ophthalmic Gel | Zirgan | 更昔洛韦眼用凝胶 | M | 5g:7.5mg | 3.83（3.62，3.96） | 0.51/mg | 5 | N |
| S01GX05 | Lodoxamide tromethamine | Alomide Ophthalmic Solution | 洛度沙胺氨丁三醇滴眼液 | M | 0 | 0 | 0 | 0 | N |
| S01LA04 | Ranibizumab (Genetical Recombination) | Lucentis | 雷珠单抗注射液 | M | 0 | 0 | 0 | 0 | N |
| S02AA16 | Ofloxacin | Ocuflox Ophthalmic Solution | 氧氟沙星滴眼液、眼膏 | M and I | 3.5g:10.5mg | 3.33（2.25，4.17） | 0.32/mg | 6 | N |
| V03AB23 | Acetylcysteine | Acetadote | 乙酰半胱氨酸注射液 | M | 20ml:4000mg | 5.03（4.87，5.27） | 0.001/mg | 4 | N |
| V03AB33 | Hydroxocobalamin | Cyanokit | 盐酸羟钴胺注射液 | M | 0 | 0 | 0 | 0 | N |
| V03AC02 | Deferiprone | Ferriprox | 去铁酮片 | I | 500mg | 2.97（2.84，3.04） | 0.006/mg | 2 | N |
| V03AC03 | Deferasirox | Exjade | 地拉罗司分散片 | I | 125mg | 11.59（11.59，11.59） | 0.09/mg | 1 | N |
| V03AF01 | Mesna | Mesnex | 美司钠注射液 | M and I | 4ml:400mg | 2.26（1.75，3.68） | 0.006/mg | 6 | Y |
| V03AF02 | Dexrazoxane | Zinecard | 注射用右丙亚胺 | M | 250mg | 71.39（66.67，74.84） | 0.29/mg | 8 | N |
| V03AF03 | Leucovorin | Leucovorin Calcium | 亚叶酸钙注射液 | M and I | 10ml:100mg | 54.71（32.31，90.40） | 0.55/mg | 7 | Y |
| V03AF05 | Amifostine | Ethyol | 注射用氨磷汀 | M | 500mg | 58.16（46.79，76.92） | 0.12/mg | 8 | N |

M: a drug was manufactured in China. I: a drug was imported in China. EM: Essential Medicine. Y= Yes, N= NO. *Procurement prices were obtained from the centralized pharmaceutical bidding system of government web-platforms of 31 provinces in 2011.
