# Supplementary material for: The availability and affordability of orphan drugs for rare diseases in China
Source: Orphanet J Rare Dis. 2016 Feb 27;11:20. doi: 10.1186/s13023-016-0392-4 (PMC4769558; doi:10.1186/s13023-016-0392-4)
Supplement: Additional file 6: Table S6. — Unauthorized list of 35 orphan drugs for 14 rare diseases in the China market. (DOC 59 kb) [file 13023_2016_392_MOESM6_ESM.doc]

**Additional file 6:** Table S6. Unauthorized list of 35 orphan drugs for 14 rare diseases in the China market

| **ATC Code** | **Generic name ( Brand name )** | **Indication** | **Year marketing authorization received** ^★^ | | |
| --- | --- | --- | --- | --- | --- |
|  |  |  | US | EU | Japan |
| A16AB01 | Alglucerase (Ceredase) * **P** | **GD** | 1991 | -- | 1996 |
| A16AX06 | Miglustat capsule (Zavesca) * |  | 2003 | 2002 | 2012 |
| A16AB10 | Velaglucerase alfa (Vpriv) * **P** |  | 2010 | 2010 | --D |
| A16AB11 | Taliglucerase alfa (Elelyso) * |  | 2012 | --D | -- |
| A16AX07 | Sapropterin hydrochloride (Biopten) | **PKU** | -- | -- | 2008 |
| -- | Antihemophilic factor (recombinant) (Kogenate) | **HEM** | 1993 | -- | -- |
| -- | Human antihemophilic factor (Humate-P ) |  | 1999 | -- | -- |
| -- | Recombinant antihemophilic factor (ReFacto) |  | 2000 | -- | -- |
| B01AC21 | Treprostinil inhalational (Tyvaso) | **PAH** | 2009 | -- | -- |
| B01AC09 | Epoprostenol sodium (Flolan) * P |  | 1995 | -- | 2004 |
| R07AX01 | Nitric oxide gas inhalational (Inomax/Inoflo) * P |  | 1999 | --,◊ | 2008 |
| B06AC02 | Icatibant (Firazyr) * P | **HAE** | 2011 | 2008 | -- |
| B06AC03 | Ecallantide (Kalbitor®) * |  | 2009 | -- | --D |
| -- | C1 Esterase inhibitor (Cinryze) |  | 2008 | -- | -- |
| -- | C1 Esterase inhibitor (Berinert P) |  | 2009 | -- | -- |
| L01XE14 | Bosutinib (Bosulif) * | **CML** | 2012 | --D | -- |
| L01XX40 | Omacetaxine mepesuccinate (Synribo) * |  | 2012 | -- | -- |
| L01XE24 | Ponatinib (Iclusig) * P |  | 2012 | -- | -- |
| L01XX02 | Erwinia L-Asparaginase (Erwinase) ^∆^ | **ALL** | 2011 | --D | -- |
| L01BB06 | Clofarabine (Clolar/Evoltra）* P |  | 2004 | 2006 | --D |
| L01BB07 | Nelarabine (Arranon/Atriance) * P |  | 2005 | 2007 | 2007 |
| L01CA02 | Vincristine sulfateliposome injection (Marqibo) |  | 2012 | --D | -- |
| L01XE24 | Ponatinib (Iclusig) *P |  | 2012 | -- | -- |
| L01XC05 | Gemtuzumab ozogamicin (Mylotarg) * P | **AML** | 2000 | --D | 2005 |
| L03AA02 | Filgrastim(Neupogen) |  | 1998 | --,◊ | -- |
| L03AA09 | Sargramostim(Leukine) |  | 1995 | -- | -- |
| L01BC07 | Azacitidine(Vidaza) * **P** |  | --D | 2008 | --• |
| -- | Tamibarotene (Amnolake) | **APL** | --D | -- | 2005 |
| V10XX02 | Ibritumomab Tiuxetan (Zevalin) | **NHL** | 2002 | --,◊ | -- |
| L01XX38 | Vorinostat (Zolinza) * P |  | 2006 | -- | 2011 |
| V10XA53 | Tositumomab And Iodine I 131 Tositumomab (Bexxar) |  | 2003 | --D | -- |
| L01AA09 | Bendamustine hydrochloride (Treakisym) |  | -- | -- | 2010 |
| L03AC01 | Aldesleukin(Proleukin) | **RCC** | 1992 | -- | -- |
| L01XE09 | Temsirolimus (Torisel) *P |  | 2007 | 2007 | -- |
| R07AA01 | Colfosceril palmitate (Exosurf Neonatal ) * P | **PIRDS** | 1990 | -- | -- |

^★^: Drugs were approved as an orphan drug; ^*^: NME, new molecular entity as defined by the FDA; ^∆^: NBE, new biologic entity as defined by the FDA; D: Orphan drug designation authorized; P: The drug received FDA priority drug review. ^◊^: The drug was intended for rare diseases in Europe with European market authorization without priority designation of orphan drug in Europe; ^♦^: The drug had a generic equivalent in China.
